# Use and Performance of the STOP-Bang Questionnaire for Obstructive Sleep Apnea Screening Across Geographic Regions: A Systematic Review and Meta-Analysis

**DOI:** 10.1001/jamanetworkopen.2021.1009

**Published:** 2021-03-08

**Authors:** Bianca Pivetta, Lina Chen, Mahesh Nagappa, Aparna Saripella, Rida Waseem, Marina Englesakis, Frances Chung

**Affiliations:** 1 Department of Anesthesia and Pain Management, Toronto Western Hospital, University Health Network, University of Toronto, Toronto, Ontario, Canada; 2 Department of Anesthesia and Perioperative Medicine, Western University, London, Ontario, Canada; 3 Library and Information Services, University Health Network, Toronto, Ontario, Canada

## Abstract

**Importance:**

Obstructive sleep apnea (OSA) is a highly prevalent global health concern and is associated with many adverse outcomes for patients.

**Objective:**

To evaluate the utility of the STOP-Bang (snoring, tiredness, observed apnea, blood pressure, body mass index, age, neck size, gender) questionnaire in the sleep clinic setting to screen for and stratify the risk of OSA among populations from different geographical regions.

**Data Sources and Study Selection:**

MEDLINE, MEDLINE In-process, Embase, EmCare Nursing, Cochrane Central Register of Controlled Trials, Cochrane Database of Systematic Reviews, PsycINFO, Journals@Ovid, Web of Science, Scopus, and CINAHL electronic databases were systematically searched from January 2008 to March 2020. This was done to identify studies that used the STOP-Bang questionnaire and polysomnography testing in adults referred to sleep clinics.

**Data Extraction and Synthesis:**

Clinical and demographic data were extracted from each article independently by 2 reviewers. The combined test characteristics were calculated using 2 × 2 contingency tables. Random-effects meta-analyses and metaregression with sensitivity analyses were performed. The Preferred Reporting Items for Systematic Review and Meta-analyses (PRISMA) guideline was followed.

**Main Outcomes and Measures:**

The combined test characteristics and area under summary receiver operating characteristic curves (AUCs) were used to compare STOP-Bang questionnaire accuracy with polysomnography testing.

**Results:**

A total of 47 studies with 26 547 participants (mean [SD] age, 50 [5] years; mean [SD] body mass index, 32 [3]; 16 780 [65%] men) met the criteria for the systematic review. Studies were organized in different geographic regional groups: North America, South America, Europe, Middle East, East Asia, and South or Southeast Asia. The prevalence rates for all OSA, moderate to severe OSA, and severe OSA were 80% (95% CI, 80%-81%), 58% (95% CI, 58%-59%), and 39% (95% CI, 38%-39%), respectively. A STOP-Bang score of at least 3 had excellent sensitivity (>90%) and high discriminative power to exclude moderate to severe and severe OSA, with negative predictive values of 77% (95% CI, 75%-78%) and 91% (95% CI, 90%-92%), respectively. The diagnostic accuracy of a STOP-Bang score of at least 3 to detect moderate to severe OSA was high (>0.80) in all regions except East Asia (0.52; 95% CI, 0.48-0.56).

**Conclusions and Relevance:**

The results of this meta-analysis suggest that the STOP-Bang questionnaire can be used as a screening tool to assist in triaging patients with suspected OSA referred to sleep clinics in different global regions.

## Key Points

**Question:**

Is the STOP-Bang (snoring, tiredness, observed apnea, blood pressure, body mass index, age, neck size, gender) questionnaire a valid obstructive sleep apnea screening tool for patients referred to sleep clinics in different geographical populations?

**Findings:**

This systematic review and meta-analysis of 47 studies including 26 547 individuals found that the STOP-Bang questionnaire has adequate sensitivity and diagnostic accuracy for detecting moderate to severe obstructive sleep apnea across geographic regions.

**Meaning:**

These findings suggest that the STOP-Bang questionnaire can be used as a screening tool in different geographical regions for triaging patients suspected of having obstructive sleep apnea who are referred to sleep clinics.

## Teaser

This systematic review and meta-analysis evaluates the utility of the STOP-Bang (snoring, tired, observed apnea, blood pressure, body mass index, age, neck size, gender) questionnaire in the sleep clinic setting to screen for and stratify the risk of obstructive sleep apnea among populations from different geographical regions.

## Introduction

Obstructive sleep apnea (OSA) is an increasingly prevalent health condition globally. Its prevalence varies depending on geographic and demographic factors.^1^ Nevertheless, worldwide, an estimated 425 million individuals aged 30 to 69 years have moderate to severe OSA,^2^ and in the general adult population, 80% to 90% of OSA is untreated and undiagnosed.^3^ Unrecognized OSA is a significant health concern,^4^ associated with various diseases,^5,6^ public safety hazards,^7^ and all-cause mortality.^8^ Thus, it is imperative to consider strategies focusing on early diagnosis and treatment of OSA.

The criterion-standard test for OSA diagnosis is laboratory polysomnography (PSG), but it is costly and inconvenient. Home sleep apnea testing (HSAT) is an acceptable alternative; however, accessibility is lacking in resource-limited areas. Since waitlists are long for patient assessment at a sleep clinic, an effective screening tool is necessary to triage patients.^9^

The STOP-Bang questionnaire was developed as an OSA screening tool consisting of 4 self-reportable (STOP: snoring, tiredness, observed apnea, and high blood pressure) and 4 demographic (Bang: body mass index [BMI; calculated as weight in kilograms divided by height in meters squared], age, neck circumference, and gender) items. In the initial validation study, at a score of at least 3, the STOP-Bang questionnaire demonstrated a sensitivity of 84%, 93%, and 100% to detect all OSA (apnea-hypopnea index [AHI] ≥5), moderate OSA (AHI ≥15), and severe OSA (AHI ≥30), respectively.^10^ Because of its high diagnostic accuracy, ease of use, and clear thresholds for risk stratification,^10,11^ the STOP-Bang questionnaire has been used worldwide. Two STOP-Bang questionnaire items, BMI and neck circumference, are influenced by region-specific body characteristics, which may affect the performance of STOP-Bang questionnaire in different geographic areas. The objective of this systematic review and meta-analysis was to determine the utility of the STOP-Bang questionnaire as an OSA screening tool to assist in triaging patients referred to sleep clinics in different global regions.

## Methods

### Literature Search and Data Sources

The protocol of this systematic review and meta-analysis was registered in the International Prospective Register of Systematic Reviews (PROSPERO) (CRD42020196952) and followed the Preferred Reporting Items for Systematic Reviews and Meta-analyses (PRISMA) reporting guideline.^12^ One of us, a medical information specialist (M.E.), designed a literature search strategy using free-text and index terms (ie, *stop-bang* or *stopbang*) and systematically searched the following databases from January 2008 to March 2020 with no language restrictions: MEDLINE, MEDLINE In-process, Embase, EmCare Nursing, Cochrane Central Register of Controlled Trials, Cochrane Database of Systematic Reviews, PsycINFO and Journals@Ovid with full-text searching using the Ovid search interface; Web of Science (Clarivate Analytics), Scopus (Elsevier), and CINAHL. A Web of Science citation search was run on the initial STOP-Bang validation article to capture publications in which it has been cited.^10^ A manual citation search was performed. Continued literature surveillance was done through August 2020. The electronic search strategy appears in eTable 1 in the Supplement.

### Inclusion Criteria and Study Selection

Two of us (B.P. and L.C.) independently screened titles and abstracts of identified studies. After initial exclusion, full-text articles were assessed for the following inclusion criteria: (1) assessment of the STOP-Bang questionnaire to screen for OSA in adults (age ≥18 years); (2) patients referred to sleep clinic; (3) laboratory PSG or HSAT results confirmed the OSA diagnosis; and (4) AHI or respiratory disturbance index (RDI) was used to diagnose and grade the severity of OSA. Exclusion criteria were (1) pregnant populations; (2) use of a modified STOP-Bang questionnaire; (3) no analysis of test characteristics at a STOP-Bang score of at least 3; and (4) inadequate description of methods (ie, no report of PSG device used and OSA diagnosed without an AHI cutoff) or insufficient data for meta-analysis. Disagreements were resolved by discussion and consensus among 4 of us (B.P., L.C., M.N., and F.C.).

### Data Extraction and Synthesis

Two of us (B.P. and L.C.) independently extracted clinical and demographic data using a predesigned form. Internal and external validity of the included studies was assessed independently according to the Cochrane Methods group’s guidelines on screening and diagnostic tests.^13^ Discrepancies were addressed with another author (M.N.).

### Defining Geographic Regional Groups

Studies were organized into groups dependent on their geographical location (ie, North America, South America, Europe, Middle East, East Asia, South or Southeast Asia). Asian countries were separated into East Asia and South or Southeast Asia to match population demographic characteristics and mitigate factors affecting OSA prevalence (ie, craniofacial characteristics and body habitus differences).^2,14^

### Statistical Analysis

Meta-analysis was performed using Review Manager version 5.3 (The Cochrane Collaboration) and Stata/SE version 14.2 (StataCorp). Summary statistics were computed for all variables of interest. Mean and standard deviation were used as appropriate for descriptive statistics. When applicable, frequencies and percentages were reported. Sample size was considered when calculating the means and standard deviations for age and BMI from individual studies.

The following AHI cutoffs were adopted: all OSA (AHI ≥5), moderate to severe OSA (AHI ≥15), and severe OSA (AHI ≥30). A STOP-Bang score of 3 or greater was adopted as a threshold. A 2 × 2 contingency table was reconstructed for each AHI cutoff at a STOP-Bang score of 3 or greater for each study. The random-effects bivariate analysis model was used to combine results from individual studies to obtain the following log-transformed summary estimates with 95% CIs: sensitivity, specificity, and log scale diagnostic odds ratio (DOR).^15,16,17^ This method analyzed paired outcomes, ie, sensitivity and specificity values from individual studies, while incorporating correlations between outcomes. For cells with a 0 value, a correction factor of 0.5 was added to prevent problems associated with sensitivity and specificity equaling.^18^ The following test characteristics were recalculated with 95% CIs: prevalence, sensitivity, specificity, positive predictive value (PPV), negative predictive value (NPV), and log scale DOR. The summary area under the receiver operating characteristic (AUC) curves were calculated by logistic regression. Combined test characteristics were recalculated for each regional group at each OSA severity cutoff. Forest plots were designed using a random-effects model, while log scale DOR and AUC curve analysis were presented to assess diagnostic ability. Heterogeneity (*I*^2^) was assessed using the χ^2^ test, with *P* < .05 indicating that heterogeneity was present.

To determine the association between STOP-Bang score and probability of moderate to severe and severe OSA, posttest probabilities were calculated as previously described and combined from studies that assessed the performance of the STOP-Bang questionnaire at scores from 3 to 8. Results were produced as a bar graph.

Metaregression and sensitivity analyses were performed on various subgroups for each factor at each OSA severity using the Open MetaAnalyst software^16^ for continuous variables (ie, age, sex, BMI, neck circumference, and prevalence) and categorical variables (sample size, study design, validation tool, OSA criteria, and regional/ethnic groups). This aimed to measure these variables’ associations with the combined estimates of sensitivity, specificity, and log scale DOR. Robustness of the combined estimates was checked by leave-one-out meta-analysis to assess individual study association with the combined estimates and heterogeneity. A 2-tailed *P* < .05 was considered statistically significant.

## Results

### Search Results and Study Characteristics

Study characteristics and demographic data are summarized in Table 1 and eTable 3 in the Supplement, respectively.^19,20,21,22,23,24,25,26,27,28,29,30,31,32,33,34,35,36,37,38,39,40,41,42,43,44,45,46,47,48,49,50,51,52,53,54,55,56,57,58,59,60,61,62,63,64,65^ The initial search yielded 3871 studies, with 162 additional studies identified through citations (eFigure 1 in the Supplement). After screening titles and abstracts, 2309 articles were excluded, and 58 full-text articles were assessed for eligibility. Forty-seven studies, with 26 547 participants, were included. Mean (SD) age and BMI among participants were 50 (5) years and 32 (3), respectively, with 16 780 (65%) men. Reasons for article exclusion are listed in eTable 2 in the Supplement. Results of internal and external study validity assessment are presented in eTable 4 and eTable 5 in the Supplement. Included studies showed low to moderate risk of bias after validity assessment and were used to answer our review question. Studies were organized into 6 groups: (1) North America (9 studies,^19,20,21,22,23,24,25,26,27^ 3507 participants); (2) South America (6 studies,^28,29,30,31,32,33^ 10 709 participants); (3) Europe (10 studies,^34,35,36,37,38,39,40,41,42,43^ 5679 participants); (4) the Middle East (11 studies,^44,45,46,47,48,49,50,51,52,53,54^ 3468 participants); (5) East Asia (4 studies,^55,56,57,58^ 1665 participants); and (6) South or Southeast Asia (7 studies,^59,60,61,62,63,64,65^ 1519 participants).

**Table 1.** Demographic Data of Patients Using STOP-Bang Questionnaire

Source	Study location	Sample size	Study type	Validation tool	Age, mean (SD), y	Men, No. (%)	Mean (SD)				
							BMI	Neck circumference, cm	STOP-Bang score	AHI, events/h	Minimum Spo_2_ (%)
**North America**											
Boynton et al,^19^ 2013	US	219	Prospective	Laboratory PSG	46 (14)	99 (45)	33 (9)	40 (5)	4 (2)	NR	NR
Farney et al,^20^ 2011	US	1424	Retrospective	Laboratory PSG	50 (15)	812 (57)	34 (8)	41 (5)	4 (2)	32 (30)	NR
McMahon et al,^21^ 2017	US	338	Retrospective	Laboratory PSG	40 (10)	257 (76)	29 (4)	40 (9)	4 (1)	13 (16)	NR
Miller et al,^22^ 2018	US	142	Cross-sectional	HSAT (ApneaLink Air) and Laboratory PSG	55 (15)	74 (52)	NR	NR	NR	NR	NR
Mou et al,^23^ 2019	US	935	Prospective	Laboratory PSG	NR	533 (57)	36 (10)	NR	5 (2)	NR	NR
Orbea et al,^24^ 2020	US	66	Retrospective	HSAT and Laboratory PSG	54 (6)	0	33 (8)	NR	3 (1)	8 (8)	NR
Pereira et al,^25^ 2013	Canada	128	Prospective	HSAT (Sandman Elite SD32+)	50 (12)	84 (66)	31 (7)	41 (4)	NR	33 (28)	NR
Sangkum et al,^26^ 2017	US	208	Cross-sectional	Laboratory PSG	53 (1)	75 (36)	37 (1)	41 (0)	NR	19 (22)	NR
Vana et al,^27^ 2013	US	47	Cross-sectional	Laboratory PSG	46 (13)	16 (34)	36 (9)	38 (5)	5 (2)	29 (23)	NR
**South America**											
Andrade et al,^28^ 2020	Brazil	35	Cross-sectional	Laboratory PSG	NR	17 (49)	33 (5)	40 (5)	NR	36 (27)	NR
Baldini et al,^29^ 2017	Argentina	327	Retrospective	Laboratory PSG	50	170 (52)	39	44	NR	NR	NR
Duarte et al,^30^ 2017	Brazil	456	Prospective	Laboratory PSG	43 (13)	292 (64)	32 (8)	41 (4)	4 (2)	25 (25)	NR
Duarte et al,^31^ 2020	Brazil	7377	Cross-sectional	Laboratory PSG	46 (15)	3984 (54)	33 (8)	41 (5)	NR	28 (28)	82 (9)
Saldias Peñafiel et al,^32^ 2018	Chile	1050	Prospective	HSAT (Embletta Gold, Embletta MPR)	56 (15)	714 (68)	31 (6)	42 (4)	NR	26 (22)	79 (10)
Saldias Peñafiel et al,^33^ 2019	Chile	1464	Prospective	Laboratory PSG	54 (15)	951 (65)	31 (6)	41.6 (4)	NR	NR	NR
**Europe**											
Bille et al,^34^ 2015	Denmark	43	Prospective	HSAT	54 (14)	34 (79)	29	NR	5	13	NR
Christensson et al,^35^ 2018	Sweden	449	Prospective	HSAT (Embletta, NOX T3)	54 (14)	274 (61)	30 (6)	NR	4 (1)	12 (15)	NR
Cowan et al,^36^ 2014	UK	129	Prospective	HSAT (SOMNOmedics)	49 (11)	83 (64)	33 (2)	NR	NR	NR	NR
Kørvel-Hanquist et al,^37^ 2018	Denmark	208	Prospective	HSAT (NOX T3)	53 (13)	152 (73)	35 (8)	NR	5 (1)	35 (20)	NR
Kuczyński et al,^38^ 2019	Poland	1123	Retrospective	Laboratory PSG	52 (12)	842 (75)	32 (6)	NR	NR	20 (6)	NR
Pataka et al,^39^ 2014	Greece	1853	Retrospective	Laboratory PSG	52 (14)	1371 (74)	33 (7)	42 (4)	5 (2)	33 (27)	NR
Pataka et al,^40^ 2019	Greece	700	Retrospective	Laboratory PSG	60 (11)	455 (65)	35 (8)	42 (8)	5 (2)	34 (24)	NR
Pataka et al,^41^ 2020	Greece	700	Prospective	Laboratory PSG	53 (14)	350 (50)	30 (9)	39 (11)	4 (1)	25 (24)	NR
Rebelo-Marques et al,^42^ 2018	Portugal	259	Prospective	HSAT (NOX T3, Stardust II, Embletta X100, Alice PDx); Laboratory PSG	55 (12)	184 (71)	31 (5)	41 (3)	5 (2)	39 (18)	NR
Reis et al,^43^ 2015	Portugal	215	Prospective	Laboratory PSG	54 (13)	135 (63)	29	40 (4)	4 (2)	16.7	NR
**Middle East**											
Acar et al,^44^ 2013	Turkey	110	Prospective	Laboratory PSG	44 (12)	103 (94)	30 (4)	NR	NR	28 (25)	NR
Alhouqani et al,^45^ 2015	United Arab Emirates	193	Prospective	Laboratory PSG	43 (12)	151 (78)	35 (9)	40 (4)	NR	35 (31)	78 (13)
Amra et al,^46^ 2018	Iran	400	Cross-sectional	Laboratory PSG	50 (10)	236 (59)	32 (7)	41 (3)	NR	NR	NR
Arslan et al,^47^ 2020	Turkey	1003	Cross-sectional	Laboratory PSG	51 (11)	702 (70)	NR	NR	NR	NR	NR
Avincsal et al,^48^ 2017	Turkey	162	Retrospective	Laboratory PSG	50 (1)	113 (70)	34 (0)	41 (0)	NR	39 (63)	NR
BaHammam et al,^49^ 2015	Saudi Arabia	100	Prospective	Laboratory PSG	47 (14)	61 (61)	34 (8)	38 (4)	4 (2)	50 (37)	NR
Bingol et al,^50^ 2016	Turkey	196	Prospective	Laboratory PSG	NR	90 (46)	NR	NR	NR	NR	NR
El-Sayed,^51^ 2012	Egypt	234	Prospective	Laboratory PSG	50 (11)	199 (85)	38 (10)	42 (4)	6 (2)	46 (33)	NR
Kashaninasab et al,^52^ 2017	Iran	250	Cross-sectional	Laboratory PSG	48 (12)	190 (76)	NR	40 (4)	NR	44 (3)	NR
Mergen et al,^53^ 2019	Turkey	217	Retrospective	Laboratory PSG	50 (10)	158 (73)	36 (4)	43 (3)	NR	NR	NR
Sadeghniiat-Haghighi et al,^54^ 2015	Iran	603	Cross-sectional	Laboratory PSG	46 (13)	452 (75)	29 (6)	40 (4)	4 (1)	NR	70 (28)
**East Asia**											
Byun et al,^55^ 2020	South Korea	778	Cross-sectional	Laboratory PSG	49 (13)	591 (76)	27 (4)	38 (4)	NR	28 (39)	80 (11)
Hu et al,^56^ 2019	China	196	Prospective	Laboratory PSG	NR	161 (82)	27 (4)	40 (4)	4 (2)	48 (22)	NR
Luo et al,^57^ 2014	China	212	Prospective	Laboratory PSG	45 (12)	189 (89)	28 (4)	41 (3)	4 (1)	44 (28)	74 (13)
Peng et al,^58^ 2018	China	479	Retrospective	Laboratory PSG	49 (14)	373 (78)	26 (4)	37 (4)	3 (1)	20 (23)	80 (14)
**South or Southeast Asia**											
Abdullah et al,^59^ 2018	Malaysia	134	Cross-sectional	Laboratory PSG	41 (13)	84 (63)	NR	NR	NR	NR	NR
Banhiran et al,^60^ 2014	Thailand	303	Cross-sectional	Laboratory PSG	NR	184 (61)	NR	NR	NR	NR	NR
Chakrabarti et al,^61^ 2019	India	80	Retrospective	Laboratory PSG	49 (112)	57 (71)	28 (5)	NR	5 (1)	34 (23)	NR
Loh et al,^62^ 2018	Singapore	591	Retrospective	Laboratory PSG	46 (14)	437 (74)	28 (6)	40 (4)	NR	25 (29)	NR
Ong et al,^63^ 2010	Singapore	319	Prospective	Laboratory PSG	47 (15)	226 (71)	28 (6)	40 (4)	4 (2)	26 (27)	82 (13)
Perumalsamy et al,^64^ 2017	India	62	Cross-sectional	Laboratory PSG	53 (12)	37 (60)	NR	NR	NR	NR	NR
Vulli et al,^65^ 2019	India	35	Prospective	Laboratory PSG	54 (6)	28 (80)	35 (3)	41 (1)	NR	NR	NR

Abbreviations: AHI, apnea-hypopnea index; BMI, body mass index (calculated as weight in kilograms divided by height in meters squared); HSAT, home sleep apnea testing; NR, not reported; PSG, polysomnography; Spo_2_, oxyhemoglobin saturation.

### Test Characteristics of the STOP-Bang Questionnaire in All Included Studies

**Figure 1.**
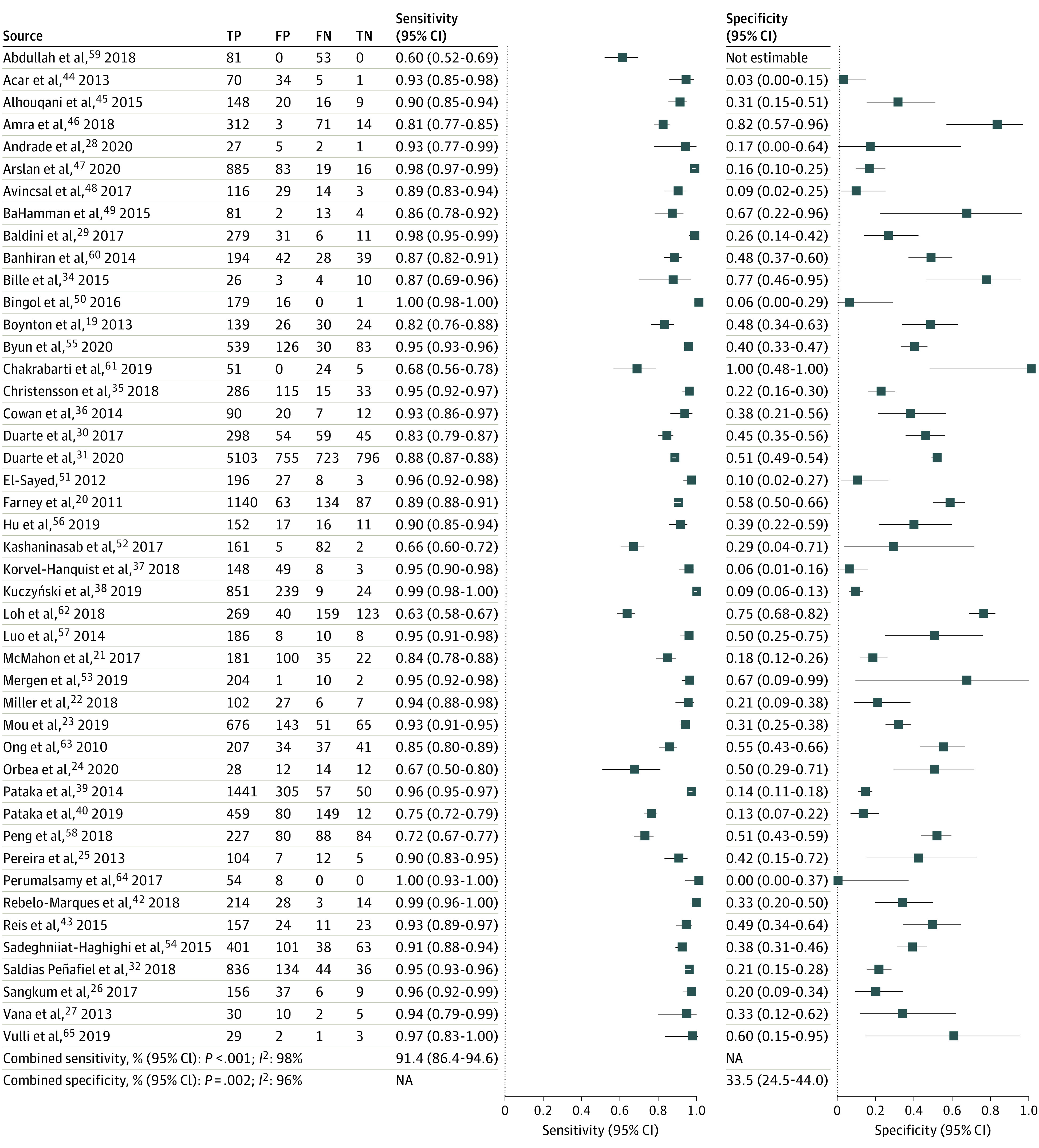
Forest Plot for Combined Sensitivity and Specificity for All Obstructive Sleep Apnea for All Included Studies in the Sleep Clinic FN indicates false-negative; FP, false-positive; NA, not applicable; TN, true-negative; TP, true-positive.

The combined test characteristics in the sleep clinic at a STOP-Bang threshold of 3 or greater are presented in Figure 1, Table 2, and eFigure 2 in the Supplement. The prevalence rates of all OSA, moderate to severe OSA, and severe OSA were 80% (95% CI, 80%-81%), 58% (95% CI, 58%-59%), and 39% (95% CI, 38%-39%), respectively. A STOP-Bang score of at least 3 had excellent sensitivity (combined sensitivity, 91.4; 95% CI, 86.4-94.6) to detect all severities of OSA. The false-negative rate was 8% (6%-11%). Moreover, the STOP-Bang questionnaire demonstrated a high discriminative power, indicated by NPVs, to exclude moderate to severe (77%, 95% CI, 75%-78%) and severe (91%, 95% CI, 90%-92%) OSA. Moderately low specificity was seen, with 28% (95% CI, 22%-34%) for moderate to severe OSA and 24% (95% CI, 19%-30%) for severe OSA.

**Table 2.** Combined Test Characteristics of a STOP-Bang Score of 3 or Greater

Test characteristic	All OSA, ie, AHI ≥5, % (95% CI)	Moderate to severe OSA, ie, AHI ≥15, % (95% CI)	Severe OSA, ie, AHI ≥30, % (95% CI)
All regions			
Studies, No. (participants, No.)	45 (24 192)	38 (23 811)	29 (17 984)
Prevalence	80 (80 to 81)	58 (58 to 59)	39 (38 to 39)
Sensitivity	92 (89 to 94)	95 (93 to 96)	97 (95 to 98)
Specificity	33 (26 to 41)	28 (22 to 34)	24 (19 to 30)
PPV	86 (85 to 86)	66 (65 to 66)	46 (45 to 47)
NPV	47 (45 to 49)	77 (75 to 78)	91 (90 to 92)
Log scale DOR (95% CI)	1.74 (0.88 to 2.59)	1.91 (1.62 to 2.21)	2.08 (1.72 to 2.43)
AUC (95% CI)	0.76 (0.72 to 0.80)	0.76 (0.72 to 0.80)	0.72 (0.68 to 0.76)
North America			
Studies, No. (participants, No.)	9 (3507)	9 (3507)	8 (3460)
Prevalence	81 (80 to 82)	54 (53 to 56)	32 (31 to 34)
Sensitivity	90 (84 to 93)	94 (93 to 96)	96 (94 to 97)
Specificity	34 (24 to 45)	24 (18 to 33)	20 (14 to 27)
PPV	86 (84 to 87)	60 (58 to 62)	36 (34 to 38)
NPV	45 (41 to 49)	78 (74 to 81)	91 (88 to 93)
Log scale DOR (95% CI)	1.21 (–0.81 to 3.24)	1.93 (1.40 to 2.47)	1.59 (1.12 to 2.07)
AUC (95% CI)	0.72 (0.68 to 0.76)	0.89 (0.85 to 0.91)	0.89 (0.86 to 0.91)
South America			
Studies, No. (participants, No.)	5 (9245)	4 (9624)	3 (8160)
Prevalence	80 (79 to 81)	58 (57 to 59)	37 (36 to 38)
Sensitivity	93 (87 to 96)	96(91 to 98)	96 (96 to 97)
Specificity	33 (21 to 48)	25(15 to 38)	30 (28 to 31)
PPV	87 (86 to 88)	66 (65 to 67)	44 (43 to 46)
NPV	52 (49 to 54)	78 (76 to 80)	93 (92 to 94)
Log scale DOR, 95% CI	4.09 (1.18 to 7.00)	1.62 (1.27 to 1.97)	2.36 (2.16 to 2.55)
AUC, 95% CI^a^	0.76 (0.72 to 0.79)	0.74	0.66
Europe			
Studies, No. (participants, No.)	9 (4979)	10 (5679)	6 (3278)
Prevalence	79 (78 to 80)	60 (58 to 61)	46 (45 to 48)
Sensitivity	95 (90 to 97)	97 (93 to 99)	99 (97 to 99)
Specificity	24 (13 to 39)	25 (14 to 40)	22 (12 to 36)
PPV	81 (80 to 82)	67 (66 to 69)	56 (54 to 58)
NPV	41 (36 to 46)	78 (75 to 81)	96 (94 to 98)
Log scale DOR, 95% CI	2.81 (1.03 to 4.59)	1.93 (1.60 to 2.26)	3.00 (1.99 to 4.01)
AUC, 95% CI	0.78 (0.74 to 0.81)	0.81 (0.78 to 0.84)	0.96 (0.93 to 0.97)
Middle East			
Studies, No. (participants, No.)	11 (3468)	7 (2545)	6 (1542)
Prevalence	87 (86 to 88)	67 (65 to 68)	48 (46 to 51)
Sensitivity	93 (87 to 96)	95 (89 to 98)	94 (85 to 98)
Specificity	24 (12 to 44)	28 (13 to 50)	33 (17 to 54)
PPV	90 (88 to 91)	71 (69 to 73)	55 (52 to 57)
NPV	30 (26 to 35)	66 (61 to 72)	78 (73 to 83)
Log scale DOR, 95% CI	0.75 (–1.27 to 2.78)	2.24 (1.11 to 3.37)	1.96 (1.48 to 2.45)
AUC, 95% CI	0.77 (0.73 to 0.80)	0.83 (0.79 to 0.86)	0.78 (0.74 to 0.81)
East Asia			
Studies, No. (participants, No.)	4 (1665)	4 (1665)	3 (887)
Prevalence	75 (73 to 77)	56 (54 to 59)	40 (37 to 43)
Sensitivity	90 (81 to 96)	93(84 to 97)	90 (87 to 93)
Specificity	44 (37 to 50)	33 (27 to 41)	30 (26 to 34)
PPV	83 (81 to 85)	64 (62 to 67)	46 (43 to 50)
NPV	56 (51 to 62)	76 (71 to 81)	82 (76 to 87)
Log scale DOR, 95% CI	1.38 (–0.19 to 2.97)	2.01 (1.24 to 2.77)	1.89 (0.74 to 3.03)
AUC. 95% CI^a^	0.56 (0.52 to 0.60)	0.52 (0.48 to 0.56)	0.41
South or Southeast Asia			
Studies, No. (participants, No.)	7 (1519)	4 (791)	3 (657)
Prevalence	75 (73 to 78)	56 (52 to 59)	34 (30 to 38)
Sensitivity	81 (68 to 89)	89 (73 to 96)	96(93 to 98)
Specificity	60 (46 to 73)	45 (30 to 61)	33 (28 to 37)
PPV	86 (84 to 89)	65 (61 to 69)	42 (38 to 47)
NPV	46 (41 to 51)	70 (63 to 76)	95 (89 to 98)
Log scale DOR, 95% CI	1.36 (0.21 to 2.52)	2.04 (1.12 to 2.96)	2.54 (1.91 to 3.17)
AUC, 95% CI^a^	0.76 (0.72 to 0.80)	0.70 (0.66 to 0.74)	0.89

Abbreviations: AHI, apnea-hypopnea index; AUC, area under the summary receiver operating characteristic curve; DOR, diagnostic odds ratio; NPV, negative predictive value; OSA, obstructive sleep apnea; PPV, positive predictive value.

^a^ For groups with fewer than 5 studies, 95% CIs for AUC values are not reported due to software output.

### Test Characteristics of the STOP-Bang Questionnaire for All OSA in Different Geographic Regional Groups

For all OSA, the combined prevalence ranged between 75% (95% CI, 73%-77%) in East Asia and 87% (95% CI, 86%-88%) in the Middle East (Table 2; eFigure 3 and eTable 6 in the Supplement). The combined sensitivities were excellent in all regions (>90%) and combined specificity values were moderately low, except in South or Southeast Asia (sensitivity: 81%; 95% CI, 68%-89%; specificity: 60%; 95% CI, 46%-73%). Similarly, PPVs were consistently high among all regional groups, ranging between 81% (95% CI, 80%-82%) in Europe and 87% (95% CI, 86%-88%) in South America. The AUC curves ranged between 0.72 (95% CI, 0.68-0.76) in North America and 0.78 (95% CI, 0.74-0.81) in Europe, with an exception in East Asia (0.56; 95% CI, 0.52-0.60).

### Test Characteristics of the STOP-Bang Questionnaire for Moderate to Severe OSA in Different Geographic Regional Groups

For moderate to severe OSA, the STOP-Bang questionnaire demonstrated an excellent combined sensitivity (>93%; eg, North America, 94%; 95% CI, 93%-96%) and low specificity (<33%) in North America, South America, Europe, and the Middle East. In East Asian, sensitivity was 93% (95% CI, 84%-97%); in South or Southeast Asia, sensitivity was 89% (95% CI, 73%-96%). In South or Southeast Asia, specificity was 45% (95% CI, 30%-61%) (Table 2; eFigure 3 and eTable 6 in the Supplement). A STOP-Bang score of at least 3 had a moderate discriminative power to exclude moderate to severe OSA, with NPVs ranging between 66% (95% CI, 65%-67%) in South America and 78% (95% CI, 74%-81%) in North America. DOR values were comparable across groups for moderate to severe OSA, ranging from 1.62 (95% CI, 1.27-197) in South America to 2.24 (95% CI, 1.11-3.37) in the Middle East. The diagnostic accuracy of a STOP-Bang score of at least 3 to detect moderate to severe OSA indicated by the AUC curve was high in North America, Europe, and the Middle East (eg, North America: 0.89; 95% CI, 0.85-0.91) but lower in the East Asian (0.52; 95% CI, 0.48-0.56) and South or Southeast Asian (0.70; 95% CI, 0.66-0.74) groups.

### Test Characteristics of the STOP-Bang Questionnaire for Severe OSA in Geographic Regional Groups

The STOP-Bang questionnaire demonstrated an excellent combined sensitivity, ranging between 90% (95% CI, 87%-93%) in East Asia and 99% (95% CI, 97%-99%) in Europe, with lower combined specificities (Table 2; eFigure 3 and eTable 6 in the Supplement). The STOP-Bang questionnaire demonstrated a high discriminative power to exclude severe OSA in all regional groups, as NPVs ranged between 82% (95% CI, 76%-87%) in East Asia and 96% (95% CI, 94%-98%) in Europe. The AUC curve value was highest in the European group (0.96, 95% CI, 0.93-0.97).

### Metaregression and Sensitivity Analysis of Various Subgroups

Metaregression and sensitivity analysis of continuous variables slightly changed the combined estimates but did not affect overall inference of the results (Table 3; eTable 7 in the Supplement). Similarly, analysis of the categorical variables slightly changed the combined estimates but did not impact the final inference of our results. Leave-one-out meta-analysis showed no individual study greatly affected the results.

**Table 3.** Metaregression and Sensitivity Analysis of the STOP-Bang Questionnaire for Various Subgroups According to the Severity of OSA

Covariate (studies, No.)	Sensitivity				Log scale diagnostic odds ratio			
	Point estimate (95% CI)	*I*^2^, %	Coefficient (SE)	*P* value	Point estimate (95% CI)	*I*^2^, %	Coefficient (SE)	*P* value
**Apnea-hypopnea index ≥5 (45)**								
Age (42)	91.0 (85.6 to 94.5)	98	0.064 (0.057)	.26	1.72 (0.82 to 2.62)	98	0.115 (0.105)	.28
Gender (45)	91.4 (86.4 to 94.6)	98	–0.010 (0.014)	.46	1.74 (0.88 to 2.59)	97	–0.022 (0.024)	.36
BMI (41)	91.1 (85.7 to 94.6)	98	0.109 (0.075)	.15	1.71 (0.79 to 2.64)	98	0.171 (0.142)	.23
Neck circumference (34)	90.2 (83.6 to 94.3)	98	0.34 (0.191)	.07	1.52 (0.47 to 2.57)	98	0.505 (0.378)	.18
Sample size								
>200 (30)	89.6 (82.2 to 94.1)	98	–0.425 (0.482)	.38	1.28 (0.25 to 2.31)	98	–1.453 (0.839)	.08
<200 (15)	94.1 (87.6 to 97.3)	95	NA	NA	2.77 (1.37 to 4.16)	91	NA	NA
Study type								
Prospective (19)	93.6 (87.9 to 96.7)	97	NA	NA	2.18 (0.71 to 3.64)	98	NA	NA
Retrospective (12)	85.5 (74.6 to 92.2)	98	0.779 (0.564)	.17	0.85 (–0.48 to 2.20)	97	1.646 (0.965)	.09
Cross-sectional (14)	91.9 (79.4 to 97.1)	99	–0.403 (0.594)	.50	1.87 (0.52 − 3.23)	96	–0.526 (1.033)	.61
Validation tool								
Lab PSG (39)	91.2 (85.7 to 94.8)	98	–0.990 (0.792)	.21	1.79 (0.84 to 2.73)	98	–2.732 (1.373)	.047
HSAT (6)	92.1 (80.6 to 97.1)	94	NA	NA	1.45 (–0.36 to 3.27)	92	NA	NA
OSA criteria								
AHI ≥5 (44)	91.1 (85.9 to 94.5)	98	0.734 (1.651)	.66	1.71 (0.84 to 2.58)	97	–0.311 (2.791)	.91
RDI ≥5 (1)^a^	97.9 (95.4 to 99.1)	NA	NA	NA		NA	NA	NA
Prevalence (45)	91.4 (86.4 to 94.6)	98	0.003 (0.025)	.92	1.74 (0.88 to 2.59)	97	–0.013 (0.045)	.78
Region								
North America (9)	85.7 (70.7 to 93.7)	97	NA	NA	1.21 (–0.81 to 3.24)	97	NA	NA
South America (5)	97.8 (95.0 to 99.1)	91	0.149 (0.698)	.83	4.09 (1.18 to 7.00)	98	0.011(1.225)	.99
Europe (9)	95.7 (88.4 to 98.5)	97	1.890 (0.883)	.03	2.81 (1.03 to 4.59)	96	2.529 (1.542)	.10
Middle East (11)	91.2 (73.9 to 97.4)	99	1.489 (0.733)	.04	0.75 (–1.27 to 2.78)	96	1.973 (1.284)	.12
East Asia (4)	82.7 (57.5 to 94.4)	98	0.308 (0.689)	.66	1.38 (–0.19 to 2.97)	96	–1.248 (1.231)	.31
South/Southeast Asia (7)	83.8 (72.5 to 91.0)	92	0.167 (0.871)	.85	1.36 (0.21 to 2.52)	87	0.234 (1.518)	.88
**Apnea-hypopnea index ≥15 (38)**								
Age (35)	94.4 (92.3 to 96.0)	94	0.064 (0.043)	.13	1.92 (1.60 to 2.25)	88	–0.014 (0.038)	.71
Gender (38)	94.4 (92.4 to 95.8)	93	0.021 (0.015)	.166	1.91 (1.62 to 2.21)	87	0.011 (0.015)	.44
BMI (33)	94.4 (92.4 to 95.9)	93	0.025 (0.053)	.65	1.95 (1.62 to 2.28)	88	–0.007 (0.049)	.89
Neck circumference (26)	94.3 (92.1 to 95.9)	93	0.040 (0.048)	.41	1.96 (1.58 to 2.34)	91	0.018 (0.054)	.73
Sample size								
>200 (27)	94.9 (92.9 to 96.4)^94^	94	0.345 (0.416)	.41	1.93 (1.60 to 2.26)	89	–0.029 (0.412)	.94
<200 (11)	92.5 (84.8 to 96.5)	87	NA	NA	1.90 (1.19 to 2.61)	68	NA	NA
Study type								
Prospective (19)	95.5 (92.5 to 97.3)	91	NA	NA	1.95 (1.70 to 2.20)	96	NA	NA
Retrospective (9)	93.2 (87.2 to 96.5)	95	–0.022 (0.405)	.96	1.51 (0.44 to 2.59)	96	–0.037 (0.393)	.92
Cross-sectional (10)	93.5 (87.6 to 96.7)	95	–0.527 (0.534)	.32	2.01 (1.77 to 2.24)	25	–0.700 (0.526)	.18
Validation tool								
Lab PSG (33)	94.0 (91.7 to 95.6)	94	0.418 (0.720)	.56	1.89 (1.57 to 2.20)	88	–0.106 (0.710)	.88
HSAT (5)	96.6 (93.5 to 98.3)	24	NA	NA	2.04 (1.41 to 2.67)	0	NA	NA
OSA criteria								
AHI≥5 (37)	94.1 (92.1 to 95.7)	93	2.652 (1.255)	.04	1.89 (1.60 to 2.19)	87	1.827 (1.227)	.14
RDI≥5 (1)^a^	99.1 (96.4 to 99.8)^-^	NA	NA	NA	NA	NA	NA	NA
Prevalence (38)	94.4 (92.4 to 95.8)	93	0.005 (0.018)	.76	1.91 (1.62 to 2.21)	87	0.015 (0.015)	.33
Region								
North America (9)	93.5 (91.4 to 95.1)	36	NA	NA	1.62 (1.27 to 1.97)	36	NA	NA
South America (4)	95.4 (90.6 to 97.8)	93	1.354 (0.670)	.04	1.93 (1.60 to 2.26)	48	0.174 (0.647)	.79
Europe (10)	96.5 (91.8 to 98.5)	96	0.810 (0.701)	.25	2.24 (1.11 to 3.37)	95	–0.017 (0.660)	.98
Middle East (7)	94.9 (86.7 to 98.2)	95	1.362 (0.689)	.048	2.01 (1.24 to 2.77)	78	0.585 (0.662)	.38
East Asia (4)	93.2 (78.4 to 98.1)	95	0.904 (0.574)	.12	2.04 (1.12 to 2.96)	87	0.087(0.565)	.88
South/Southeast Asia (4)	88.5 (66.8 to 96.7)	93	0.612 (0.679)	.37	1.93 (1.40 to 2.47)	28	0.655 (1.831)	.91
**Apnea-hypopnea index ≥30 (29)**								
Age (26)	95.6 (93.0 to 97.2)^89^	89	0.053 (0.052)	.31	2.07 (1.67 to 2.47)	79	0.031 (0.044)	.49
Gender (29)	95.7 (93.5 to 97.1)	88	0.029 (0.015)	.05	2.08 (1.72 to 2.43)	77	0.013 (0.013)	.32
BMI (26)	95.9 (93.8 to 97.3)	86	0.094 (0.065)	.15	2.08 (1.69 to 2.48)	79	0.057 (0.059)	.33
Neck circumference (23)	95.8 (93.3 to 97.3)	90	0.600 (0.124)	<.001	2.15 (1.75 to 2.55)	80	0.340 (0.128)	.008
Sample size								
>200 (20)	96.4 (94.3 to 97.8)	89	0.013 (0.977)	.28	2.20 (1.77 to 2.63)	82	–0.086 (0.359)	.81
<200 (9)	92.9 (84.1 to 97.0)	78	NA	NA	1.72 (1.26 to 2.18)	0	NA	NA
Study type								
Prospective (15)	96.5 (93.4 to 98.2)	78	NA	NA	2.12 (1.81 to 2.44)	NA	NA	NA
Retrospective (8)	94.2 (86.1 to 97.7)	93	–0.250 (0.416)	.55	1.78 (0.74 to 2.81)	93	–0.175 (0.278)	.53
Cross-sectional (6)	95.8 (89.3 to 98.4)	92	–0.181 (0.415)	.66	2.31 (2.13 to 2.50)	0	0.235 (0.304)	.44
Validation tool								
Lab PSG (26)	95.5 (93.1 to 97.1)	89	–0.293 (0.815)	.72	2.08 (1.71 to 2.46)	79	–0.627 (0.708)	.38
HSAT (3)	97.2 (92.2 to 99.0)	0	NA	NA	1.19 (0.45 to 1.94)	0	NA	NA
OSA criteria								
AHI ≥5 (28)	95.5 (93.2 to 97.0)	88	3.177 (1.610)	.048	2.06 (1.70 to 2.42)	77	1.714 (1.488)	.25
RDI ≥5 (1)^a^	99.7 (95.3 to 1.0)^-^	NA	NA	NA	NA	NA	NA	NA
Prevalence (29)	95.7 (93.5 to 97.1)	88	0.008 (0.025)	.75	2.08 (1.72 to 2.43)	77	0.036 (0.020)	.07
Region								
North America (8)	94.9 (91.7 to 96.9)	48	NA	NA	1.59 (1.12 to 2.07)	33	NA	NA
South America (3)	96.3 (93.5 to 97.9)	42	1.645 (0.583)	.005	2.36 (2.16 to 2.55)	0	0.393 (0.463)	.40
Europe (6)	98.3 (96.9 to 99.1)	32	1.145 (0.583)	.049	3.00 (1.99 to 4.01)	69	0.557 (0.364)	.13
Middle East (6)	93.4 (83.8 to 97.5)	90	2.126 (0.615)	<.001	1.96 (1.48 to 2.45)	30	1.738 (0.450)	<.001
East Asia (3)	93.5 (70.5 to 98.9)	92	0.682 (0.544)	.21	1.89 (0.74 to 3.03)	83	0.187 (0.423)	.66
South/Southeast Asia (3)	96.2 (92.6 to 98.0)	0	0.995 (1.586)	.09	2.54 (1.91 to 3.17)	0	0.676 (0.460)	.14

Abbreviations: AHI, apnea-hypopnea index; BMI, body mass index; HSAT, home sleep apnea testing; NA, not applicable; OSA, obstructive sleep apnea; PSG, polysomnogram; RDI, respiratory disturbance index.

^a^ No heterogeneity or log scale diagnostic odds ratio available for groups with fewer than 5 studies.

### Predictive Performance and Predictive Probability of STOP-Bang Scores of 3 to 8

For all OSA, as the STOP-Bang score increased from 3 to 8, the specificity increased from 40.6% (95% CI, 39.3%-42.0%) to 99.7% (95% CI, 99.0%-99.9%), while sensitivity decreased from 89.2% (95% CI, 88.7%-89.6%) to 3.6% (95% CI, 3.0%-4.3%) (eTable 8 in the Supplement). The PPVs and NPVs showed similar trends. This was similar for moderate to severe OSA and severe OSA.

**Figure 2.**
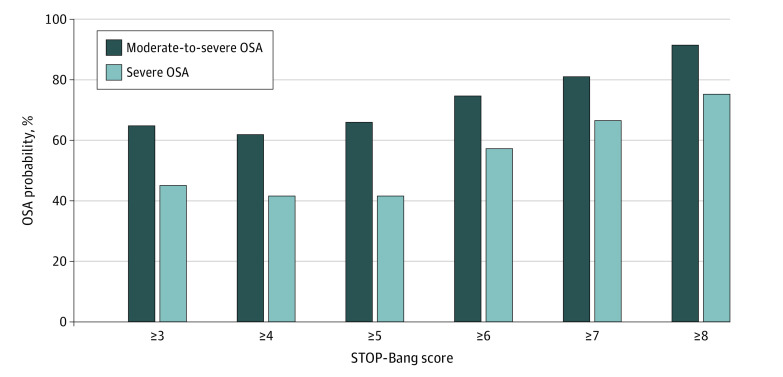
Association Between STOP-Bang Scores of 3 to 8 and Probability of Obstructive Sleep Apnea (OSA)

In all included studies, as the STOP-Bang score increased from 3 to 6, the probability of moderate to severe OSA increased from 65% to 75% (Figure 2). Similarly, the probability of having severe OSA with a STOP-Bang score of 3 was 45%, and with a score of 6, it was 57%.

## Discussion

This systematic review and meta-analysis found that the STOP-Bang questionnaire was a useful screening tool to triage patients with suspected OSA in sleep clinics in different geographic regions. Its high sensitivity would help to identify those at risk for all, moderate to severe, and severe OSA, while high NPVs would allow clinicians to rule out severe OSA in individuals with a STOP-Bang score of 2 or less. The AUC was clinically significant at each AHI threshold, indicative of its global screening utility.

The prevalence for all, moderate to severe, and severe OSA at sleep clinics globally was high at 80% (95% CI, 80%-81%), 58% (95% CI, 58%-59%), and 39% (95% CI, 38-39). Patients referred to sleep clinics are already suspected of having OSA, which explains the high prevalence and diagnostic accuracy in our study. In this setting, the STOP-Bang questionnaire fulfills the role of a screening tool that may allocate resource-limited care according to severity. While STOP-Bang scores of 3 to 5 had similar posttest probabilities (ie, 65%-66%), individuals who scored 6 or greater had a 75% posttest probability of being diagnosed with moderate to severe OSA and should be investigated more urgently. The implications of our findings are that the STOP-Bang questionnaire is a helpful tool when triaging patients who were referred to sleep clinics.

Groups were organized relative to country and geographic proximity. Asian countries were separated into East Asia and South or Southeast Asia to minimize external factors that influence OSA prevalence (ie, sleep clinic referral patterns and burden of other comorbidities)^2^ as well as population-specific anatomic and phenotypic features that influence OSA development and severity. Although OSA is prevalent globally, certain regions report higher prevalences, ie, China, the United States, Brazil, and India.^2^ These differences influence prevalence-dependent variables, such as pretest and posttest probabilities. We have addressed this by organizing studies based on geography to accurately represent OSA prevalence in each global region. Moreover, DOR values, which are independent of prevalence, were comparable across groups for moderate-to-severe OSA, ranging from 1.62 (95% CI, 1.27-197) in South America to 2.24 (95% CI, 1.11-3.37) in the Middle Eastern group.

The North American group showed a high sensitivity of 94% (95% CI, 93%-96%) and AUC of 0.89 (95% CI, 0.85-0.91) to detect moderate to severe OSA with lower specificities. Similar results were found for the South American, European, and the Middle Eastern groups. Asian groups showed lower sensitivity and AUC values at the same AHI cutoff, ie, 93% (95% CI, 84%-97%) and 0.52 (95% CI, 0.48-0.56) in the East Asian group; 89% (95% CI, 73%-96%) and 0.70 (95% CI, 0.66-0.74) in the South or Southeast Asian group; both groups had higher specificities.

Variation in the STOP-Bang questionnaire performance among these geographic groups may be explained by obesity and craniofacial characteristics. Obesity prevalence and patterns of adiposity vary among ethnic groups. In European, North American, South American, and Asian populations, AHI increases with BMI; however, BMI is most associated with OSA severity in the latter 2 regions.^66,67^ In Asian populations, higher body fat percentages and visceral fat are associated with a lower BMI.^66,68,69^ This fat distribution pattern is associated with increased tongue size and lower lung volumes that may contribute to nocturnal upper airway collapse,^70^ such that Asians present with OSA at lower BMIs and are more sensitive to BMI increases.^71^ Changing the STOP-Bang BMI cutoff to 30, closer to the obesity cutoff for Asian populations, has shown increases in sensitivity without compromising specificity for OSA detection.^57,60,61^

The craniofacial features are important in OSA pathogenesis and are unique depending on location. Chinese individuals have a smaller, narrower retropalatal airway and soft tissues with greater susceptibility to pharyngeal collapse.^72^ Differences are found between ethnic groups, such that Asian populations have more craniofacial restriction,^73^ while European groups have larger upper airway soft tissues (ie, larger tongue and parapharyngeal fat pads)^73^ with smaller airway dimensions.^14^ Similarly, North American and South American populations also showed increased neck circumference, which is associated with greater odds of a higher Mallampati class, indicative of oropharyngeal crowding, while Asian groups do not.^66^ This may explain the excellent clinical utility seen in each regional group other than the East Asian population. Further research is necessary to investigate the contribution of ethnic and genetic factors to OSA prevalence and severity.

Ideally, a diagnostic test has high sensitivity with sufficient specificity, is inexpensive, and allows for early disease identification. High sensitivity is helpful to rule out OSA and/or to prioritize patients referred to sleep clinics; however, it is an incomplete study estimate, especially in the presence of study heterogeneity and bias. False-negativity is a component of sensitivity that provides a robust summary estimate and may be considered independent of prevalence. In our meta-analysis, the false-negative rate was 8% (6%-11%). Considering specificity may improve accuracy. A moderate to low specificity at a STOP-Bang score of at least 3 may subject patients to false-positive results, increasing unnecessary costs and caseloads at sleep clinics. Nevertheless, with the goal of diagnosing and preventing mortality and morbidity associated with OSA as well as the low risk of investigating potential OSA, false-positives are of secondary importance.

### Limitations

This study has limitations. First, included studies used various laboratory PSG and HSAT devices to diagnose OSA. Although often comparable, some inconsistences may exist. Second, risk of bias for internal validity was occasionally unclear, specifically whether PSG and STOP-Bang results were masked and interpreted independent of clinical information. Third, metaregression analysis showed positive confounders contributing to differences between groups, thus enhancing combined estimates of our results. Some confounders were related to components of the STOP-Bang questionnaire (age, sex), while others were not (OSA definition). Next, although the STOP-Bang questionnaire had high NPVs for moderate to severe and severe OSA, it is possible to miss patients with OSA who have lower AHIs because of lower NPVs in patients with mild OSA. Additionally, our results are unique to the sleep clinic and do not apply to other populations owing to inflated OSA prevalence, given that patients were screened before referral for further OSA investigation. Furthermore, moderate-to-high heterogeneity exists. This may be attributed to methodological heterogeneity and variability in study location; however, all studies were grouped with efforts to unify populations. A random-effects model for meta-analysis was performed because of the suspicion of high heterogeneity. Despite these limitations, our study provides an interpretation of the available literature on the STOP-Bang questionnaire as an OSA screening tool among different global populations in the sleep clinic setting.

## Conclusions

In this study, the STOP-Bang questionnaire showed high sensitivity and NPVs at all AHI cutoffs. These findings indicate that it is a useful OSA screening tool for triaging patients in sleep clinics in various global regions.

## Notes

Supplement. **eFigure 1. **Study Flow Diagram **eFigure 2. **Forest Plot for Combined Sensitivity and Specificity for Moderate to Severe OSA and Severe OSA for All Included Studies in the Sleep Clinic **eFigure 3. **Forest Plots for Combined Sensitivity and Specificity for Various OSA Severities in Different Geographic Regional Groups **eTable 1. **Search Strategy **eTable 2. **Excluded Studies and Reasons for Exclusion **eTable 3. **Characteristics of Included Studies **eTable 4. **Appraisal of Included Studies Based on Criteria for Internal Validity **eTable 5. **Appraisal of Included Studies Based on Criteria for External Validity **eTable 6. **Tables Describing the 2 × 2 Contingency Values and Test Characteristics of Individual Studies for All OSA (AHI ≥5), Moderate to Severe OSA (AHI ≥15), and Severe OSA (AHI ≥30) **eTable 7. **Specificity Analysis of the STOP-Bang Questionnaire for Various Subgroups According to the Severity of OSA **eTable 8. **Test Characteristics of the Various STOP-Bang Score Cutoffs for the Different Severity of Apnea-Hypopnea Index in the Sleep Clinic Setting
