# Supplementary material for: Use and Performance of the STOP-Bang Questionnaire for Obstructive Sleep Apnea Screening Across Geographic Regions: A Systematic Review and Meta-Analysis
Source: JAMA Netw Open. 2021 Mar 8;4(3):e211009. doi: 10.1001/jamanetworkopen.2021.1009 (PMC7941199; doi:10.1001/jamanetworkopen.2021.1009)
Supplement: Supplement. — eFigure 1. Study Flow Diagram eFigure 2. Forest Plot for Combined Sensitivity and Specificity for Moderate to Severe OSA and Severe OSA for All Included Studies in the Sleep Clinic eFigure 3. Forest Plots for Combined Sensitivity and Specificity for Various OSA Severities in Different Geographic Regional Groups eTable 1. Search Strategy eTable 2. Excluded Studies and Reasons for Exclusion eTable 3. Characteristics of Included Studies eTable 4. Appraisal of Included Studies Based on Criteria for Internal Validity eTable 5. Appraisal of Included Studies Based on Criteria for External Validity eTable 6. Tables Describing the 2 × 2 Contingency Values and Test Characteristics of Individual Studies for All OSA (AHI ≥5), Moderate to Severe OSA (AHI ≥15), and Severe OSA (AHI ≥30) eTable 7. Specificity Analysis of the STOP-Bang Questionnaire for Various Subgroups According to the Severity of OSA eTable 8. Test Characteristics of the Various STOP-Bang Score Cutoffs for the Different Severity of Apnea-Hypopnea Index in the Sleep Clinic Setting [file jamanetwopen-e211009-s001.pdf]

Supplemental Online Content

Pivetta B, Chen L, Nagappa M, et al. Use and performance of the STOP-Bang questionnaire for obstructive sleep apnea screening across geographic regions: a systematic review and meta-analysis. *JAMA Netw Open*. 2021;4(3):e211009. doi:10.1001/jamanetworkopen.2021.1009

eFigure 1. Study Flow Diagram
eFigure 2. Forest Plot for Combined Sensitivity and Specificity for Moderate to Severe OSA and Severe OSA for All Included Studies in the Sleep Clinic
eFigure 3. Forest Plots for Combined Sensitivity and Specificity for Various OSA Severities in Different Geographic Regional Groups
eTable 1. Search Strategy
eTable 2. Excluded Studies and Reasons for Exclusion
eTable 3. Characteristics of Included Studies
eTable 4. Appraisal of Included Studies Based on Criteria for Internal Validity
eTable 5. Appraisal of Included Studies Based on Criteria for External Validity
eTable 6. Tables Describing the 2 × 2 Contingency Values and Test Characteristics of Individual Studies for All OSA (AHI ≥5), Moderate to Severe OSA (AHI ≥15), and Severe OSA (AHI ≥30)
eTable 7. Specificity Analysis of the STOP-Bang Questionnaire for Various Subgroups According to the Severity of OSA
eTable 8. Test Characteristics of the Various STOP-Bang Score Cutoffs for the Different Severity of Apnea-Hypopnea Index in the Sleep Clinic Setting

This supplemental material has been provided by the authors to give readers additional information about their work.

© 2021 Pivetta B et al. *JAMA Network Open.*

eFigure 1. Study Flow Diagram

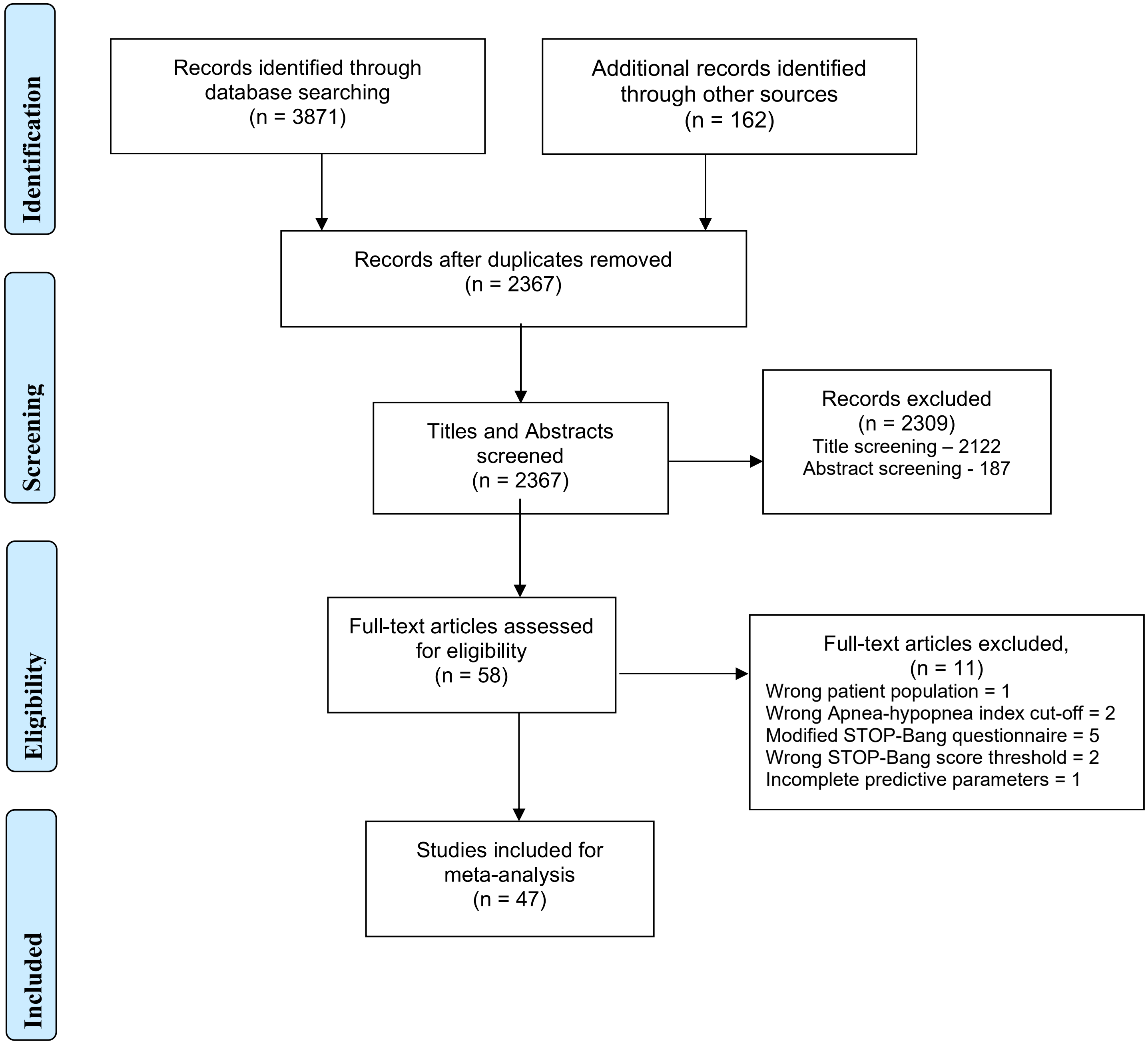

© 2021 Pivetta B et al. *JAMA Network Open.*

eFigure 2. Forest Plot for Combined Sensitivity and Specificity for Moderate to Severe OSA and Severe OSA for All Included Studies in the Sleep Clinic

A. Moderate-to-severe OSA or AHI ≥ 15

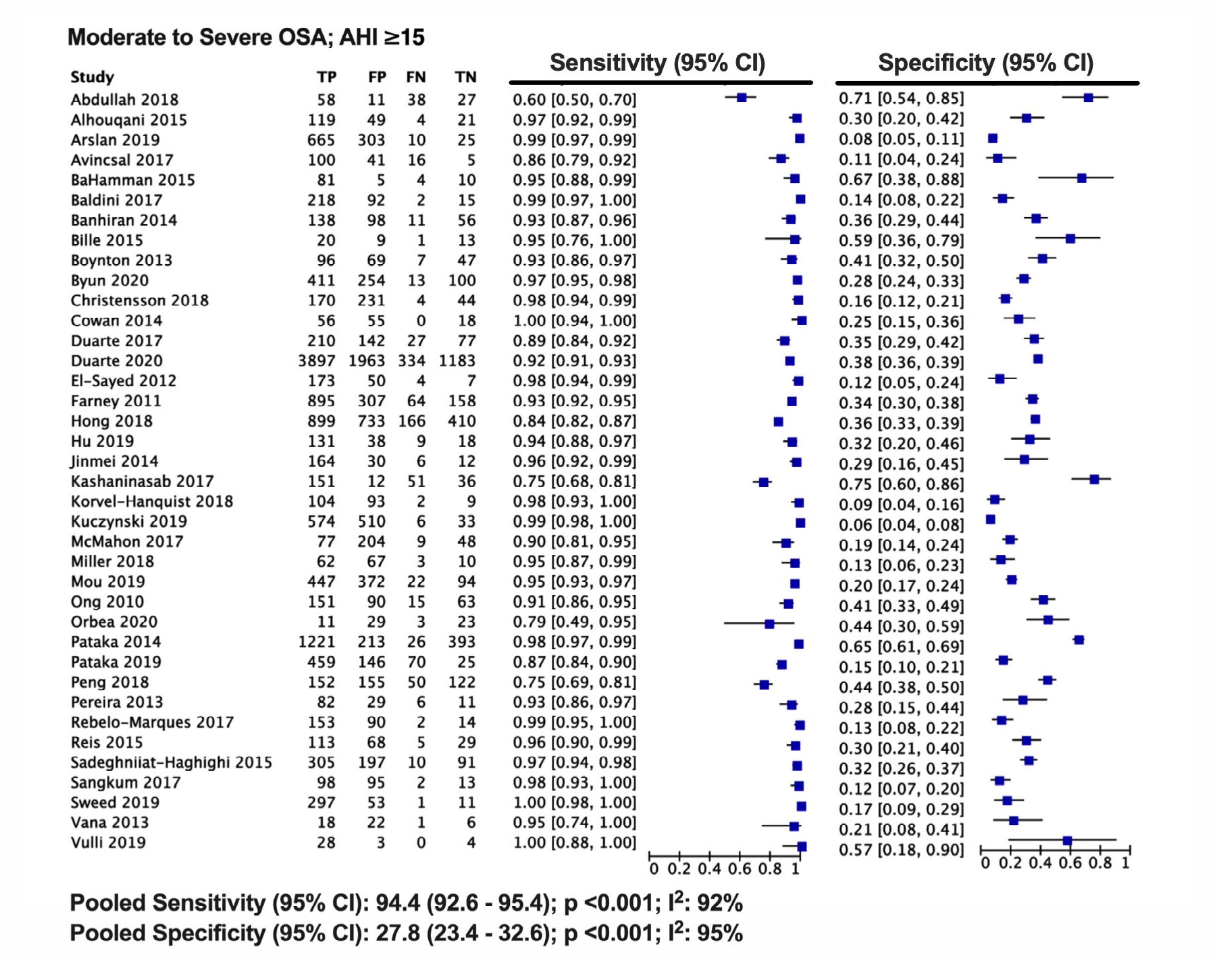

Moderate to Severe OSA; AHI ≥15

Study	TP	FP	FN	TN	Sensitivity (95% CI)	Specificity (95% CI)
Abdullah 2018	58	11	38	27	0.60 [0.50, 0.70]	0.71 [0.54, 0.85]
Alhouqani 2015	119	49	4	21	0.97 [0.92, 0.99]	0.30 [0.20, 0.42]
Arslan 2019	665	303	10	25	0.99 [0.97, 0.99]	0.08 [0.05, 0.11]
Avincsal 2017	100	41	16	5	0.86 [0.79, 0.92]	0.11 [0.04, 0.24]
BaHamman 2015	81	5	4	10	0.95 [0.88, 0.99]	0.67 [0.38, 0.88]
Baldini 2017	218	92	2	15	0.99 [0.97, 1.00]	0.14 [0.08, 0.22]
Banhiran 2014	138	98	11	56	0.93 [0.87, 0.96]	0.36 [0.29, 0.44]
Bille 2015	20	9	1	13	0.95 [0.76, 1.00]	0.59 [0.36, 0.79]
Boynton 2013	96	69	7	47	0.93 [0.86, 0.97]	0.41 [0.32, 0.50]
Byun 2020	411	254	13	100	0.97 [0.95, 0.98]	0.28 [0.24, 0.33]
Christensson 2018	170	231	4	44	0.98 [0.94, 0.99]	0.16 [0.12, 0.21]
Cowan 2014	56	55	0	18	1.00 [0.94, 1.00]	0.25 [0.15, 0.36]
Duarte 2017	210	142	27	77	0.89 [0.84, 0.92]	0.35 [0.29, 0.42]
Duarte 2020	3897	1963	334	1183	0.92 [0.91, 0.93]	0.38 [0.36, 0.39]
El-Sayed 2012	173	50	4	7	0.98 [0.94, 0.99]	0.12 [0.05, 0.24]
Farney 2011	895	307	64	158	0.93 [0.92, 0.95]	0.34 [0.30, 0.38]
Hong 2018	899	733	166	410	0.84 [0.82, 0.87]	0.36 [0.33, 0.39]
Hu 2019	131	38	9	18	0.94 [0.88, 0.97]	0.32 [0.20, 0.46]
Jinmei 2014	164	30	6	12	0.96 [0.92, 0.99]	0.29 [0.16, 0.45]
Kashaninasab 2017	151	12	51	36	0.75 [0.68, 0.81]	0.75 [0.60, 0.86]
Korvel-Hanquist 2018	104	93	2	9	0.98 [0.93, 1.00]	0.09 [0.04, 0.16]
Kuczynski 2019	574	510	6	33	0.99 [0.98, 1.00]	0.06 [0.04, 0.08]
McMahon 2017	77	204	9	48	0.90 [0.81, 0.95]	0.19 [0.14, 0.24]
Miller 2018	62	67	3	10	0.95 [0.87, 0.99]	0.13 [0.06, 0.23]
Mou 2019	447	372	22	94	0.95 [0.93, 0.97]	0.20 [0.17, 0.24]
Ong 2010	151	90	15	63	0.91 [0.86, 0.95]	0.41 [0.33, 0.49]
Orbea 2020	11	29	3	23	0.79 [0.49, 0.95]	0.44 [0.30, 0.59]
Pataka 2014	1221	213	26	393	0.98 [0.97, 0.99]	0.65 [0.61, 0.69]
Pataka 2019	459	146	70	25	0.87 [0.84, 0.90]	0.15 [0.10, 0.21]
Peng 2018	152	155	50	122	0.75 [0.69, 0.81]	0.44 [0.38, 0.50]
Pereira 2013	82	29	6	11	0.93 [0.86, 0.97]	0.28 [0.15, 0.44]
Rebelo-Marques 2017	153	90	2	14	0.99 [0.95, 1.00]	0.13 [0.08, 0.22]
Reis 2015	113	68	5	29	0.96 [0.90, 0.99]	0.30 [0.21, 0.40]
Sadeghniiat-Haghighi 2015	305	197	10	91	0.97 [0.94, 0.98]	0.32 [0.26, 0.37]
Sangkum 2017	98	95	2	13	0.98 [0.93, 1.00]	0.12 [0.07, 0.20]
Sweed 2019	297	53	1	11	1.00 [0.98, 1.00]	0.17 [0.09, 0.29]
Vana 2013	18	22	1	6	0.95 [0.74, 1.00]	0.21 [0.08, 0.41]
Vulli 2019	28	3	0	4	1.00 [0.88, 1.00]	0.57 [0.18, 0.90]

Pooled Sensitivity (95% CI): 94.4 (92.6 - 95.4); p <0.001; I^2: 92%

Pooled Specificity (95% CI): 27.8 (23.4 - 32.6); p <0.001; I^2: 95%

© 2021 Pivetta B et al. *JAMA Network Open.*

B. Severe OSA or AHI ≥ 30

Severe OSA; AHI ≥30

Study	TP	FP	FN	TN	Sensitivity (95% CI)	Specificity (95% CI)
Alhouqani 2015	85	83	2	23	0.98 [0.92, 1.00]	0.22 [0.14, 0.31]
Avincsal 2017	79	62	7	14	0.92 [0.84, 0.97]	0.18 [0.10, 0.29]
BaHamman 2015	45	9	18	28	0.71 [0.59, 0.82]	0.76 [0.59, 0.88]
Baldini 2017	161	149	0	17	1.00 [0.98, 1.00]	0.10 [0.06, 0.16]
Banhiran 2014	94	142	3	64	0.97 [0.91, 0.99]	0.31 [0.25, 0.38]
Bille 2015	10	20	0	13	1.00 [0.69, 1.00]	0.39 [0.23, 0.58]
Boynton 2013	60	105	2	52	0.97 [0.89, 1.00]	0.33 [0.26, 0.41]
Duarte 2017	124	228	6	98	0.95 [0.90, 0.98]	0.30 [0.25, 0.35]
Duarte 2020	2605	3254	107	1411	0.96 [0.95, 0.97]	0.30 [0.29, 0.32]
El-Sayed 2012	146	77	2	9	0.99 [0.95, 1.00]	0.10 [0.05, 0.19]
Farney 2011	555	647	25	197	0.96 [0.94, 0.97]	0.23 [0.21, 0.26]
Hu 2019	105	64	4	23	0.96 [0.91, 0.99]	0.26 [0.18, 0.37]
Jinmei 2014	125	69	3	15	0.98 [0.93, 1.00]	0.18 [0.10, 0.28]
Kashaninasab 2017	128	35	31	56	0.81 [0.73, 0.86]	0.62 [0.51, 0.72]
Korvel-Hanquist 2018	70	127	1	10	0.99 [0.92, 1.00]	0.07 [0.04, 0.13]
McMahon 2017	29	252	4	53	0.88 [0.72, 0.97]	0.17 [0.13, 0.22]
Miller 2018	30	99	1	12	0.97 [0.83, 1.00]	0.11 [0.06, 0.18]
Mou 2019	270	549	10	106	0.96 [0.94, 0.98]	0.16 [0.13, 0.19]
Ong 2010	108	133	5	73	0.96 [0.90, 0.99]	0.35 [0.29, 0.42]
Orbea 2020	4	35	2	25	0.67 [0.22, 0.96]	0.42 [0.29, 0.55]
Pataka 2014	889	545	9	410	0.99 [0.98, 1.00]	0.43 [0.40, 0.46]
Pataka 2019	389	211	12	88	0.97 [0.95, 0.98]	0.29 [0.24, 0.35]
Peng 2018	90	239	28	122	0.76 [0.68, 0.84]	0.34 [0.29, 0.39]
Pereira 2013	54	57	2	15	0.96 [0.88, 1.00]	0.21 [0.12, 0.32]
Rebelo-Marques 2017	80	163	0	16	1.00 [0.95, 1.00]	0.09 [0.05, 0.14]
Reis 2015	61	119	1	34	0.98 [0.91, 1.00]	0.22 [0.16, 0.30]
Sadeghniiat-Haghighi 2015	197	305	4	97	0.98 [0.95, 0.99]	0.24 [0.20, 0.29]
Sangkum 2017	59	134	1	14	0.98 [0.91, 1.00]	0.09 [0.05, 0.15]
Vulli 2019	13	18	0	4	1.00 [0.75, 1.00]	0.18 [0.05, 0.40]

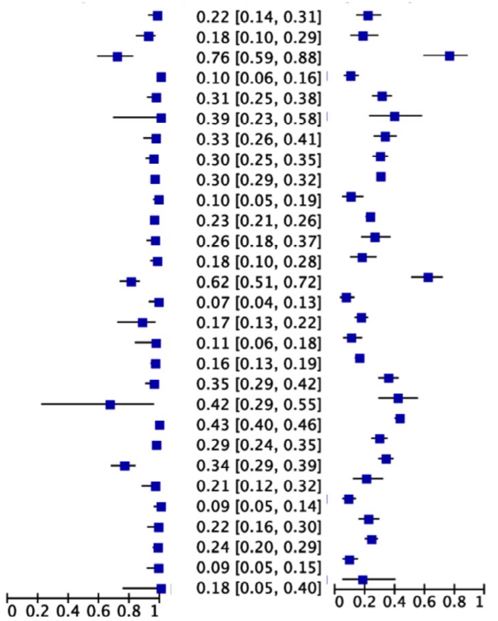

Pooled Sensitivity (95% CI): 95.7 (93.5 - 97.1); p <0.001; I^2: 88%
Pooled Specificity (95% CI): 24.6 (21.2 - 28.4); p <0.001; I^2: 93%

AHI: Apnea-hypopnea index; CI: Confidence interval; FN: False negative; FP: False positive; OSA: Obstructive sleep apnea; TN: True negative; TP: True positive.

© 2021 Pivetta B et al. *JAMA Network Open.*

eFigure 3. Forest Plots for Combined Sensitivity and Specificity for Various OSA Severities in Different Geographic Regional Groups

A. North America

All OSA (AHI ≥ 5)

Study	TP	FP	FN	TN	Sensitivity (95% CI)	Specificity (95% CI)
Boynton 2013	139	26	30	24	0.82 [0.76, 0.88]	0.48 [0.34, 0.63]
Farney 2011	1140	63	134	87	0.89 [0.88, 0.91]	0.58 [0.50, 0.66]
McMahon 2017	181	100	35	22	0.84 [0.78, 0.88]	0.18 [0.12, 0.26]
Miller 2018	102	27	6	7	0.94 [0.88, 0.98]	0.21 [0.09, 0.38]
Mou 2019	676	143	51	65	0.93 [0.91, 0.95]	0.31 [0.25, 0.38]
Orbea 2020	28	12	14	12	0.67 [0.50, 0.80]	0.50 [0.29, 0.71]
Pereira 2013	104	7	12	5	0.90 [0.83, 0.95]	0.42 [0.15, 0.72]
Sangkum 2017	156	37	6	9	0.96 [0.92, 0.99]	0.20 [0.09, 0.34]
Vana 2013	30	10	2	5	0.94 [0.79, 0.99]	0.33 [0.12, 0.62]

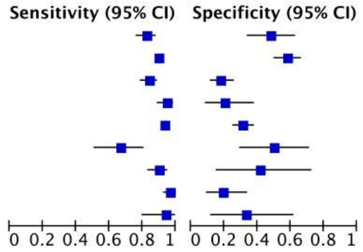

Combined Sensitivity = 0.90 (0.84-0.93); Inconsistency (I-square) = 86.6%
Combined Specificity = 0.34 (0.24-0.45); Inconsistency (I-square) = 88.0%

Moderate-to-severe OSA (AHI ≥ 15)

Study	TP	FP	FN	TN	Sensitivity (95% CI)	Specificity (95% CI)
Boynton 2013	96	69	7	47	0.93 [0.86, 0.97]	0.41 [0.32, 0.50]
Farney 2011	895	307	64	158	0.93 [0.92, 0.95]	0.34 [0.30, 0.38]
McMahon 2017	77	204	9	48	0.90 [0.81, 0.95]	0.19 [0.14, 0.24]
Miller 2018	62	67	3	10	0.95 [0.87, 0.99]	0.13 [0.06, 0.23]
Mou 2019	447	372	22	94	0.95 [0.93, 0.97]	0.20 [0.17, 0.24]
Orbea 2020	11	29	3	23	0.79 [0.49, 0.95]	0.44 [0.30, 0.59]
Pereira 2013	82	29	6	11	0.93 [0.86, 0.97]	0.28 [0.15, 0.44]
Sangkum 2017	98	95	2	13	0.98 [0.93, 1.00]	0.12 [0.07, 0.20]
Vana 2013	18	22	1	6	0.95 [0.74, 1.00]	0.21 [0.08, 0.41]

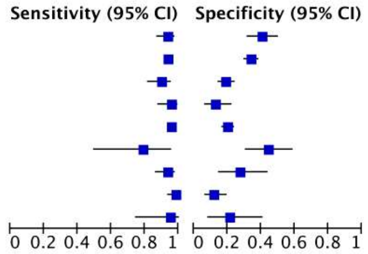

Combined Sensitivity = 0.94 (0.93-0.96); Inconsistency (I-square) = 41.7%
Combined Specificity = 0.24 (0.18-0.33); Inconsistency (I-square) = 88.7%

Severe OSA (AHI ≥ 30)

Study	TP	FP	FN	TN	Sensitivity (95% CI)	Specificity (95% CI)
Boynton 2013	61	104	2	52	0.97 [0.89, 1.00]	0.33 [0.26, 0.41]
Farney 2011	555	647	25	197	0.96 [0.94, 0.97]	0.23 [0.21, 0.26]
McMahon 2017	29	252	4	53	0.88 [0.72, 0.97]	0.17 [0.13, 0.22]
Miller 2018	30	99	1	12	0.97 [0.83, 1.00]	0.11 [0.06, 0.18]
Mou 2019	270	549	10	106	0.96 [0.94, 0.98]	0.16 [0.13, 0.19]
Orbea 2020	4	35	2	25	0.67 [0.22, 0.96]	0.42 [0.29, 0.55]
Pereira 2013	54	57	2	15	0.96 [0.88, 1.00]	0.21 [0.12, 0.32]
Sangkum 2017	59	134	1	14	0.98 [0.91, 1.00]	0.09 [0.05, 0.15]

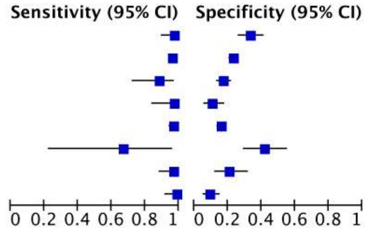

Combined Sensitivity = 0.96 (0.94-0.97); Inconsistency (I-square) = 64.3%
Combined Specificity = 0.20 (0.14-0.27); Inconsistency (I-square) = 89.0%

© 2021 Pivetta B et al. *JAMA Network Open.*

B. South America

All OSA (AHI ≥ 5)

Study	TP	FP	FN	TN	Sensitivity (95% CI)	Specificity (95% CI)
Andrade 2020	27	5	2	1	0.93 [0.77, 0.99]	0.17 [0.00, 0.64]
Baldini 2017	279	31	6	11	0.98 [0.95, 0.99]	0.26 [0.14, 0.42]
Duarte 2017	298	54	59	45	0.83 [0.79, 0.87]	0.45 [0.35, 0.56]
Duarte 2020	5103	755	723	796	0.88 [0.87, 0.88]	0.51 [0.49, 0.54]
Saldias Penafiel 2018	836	134	44	36	0.95 [0.93, 0.96]	0.21 [0.15, 0.28]

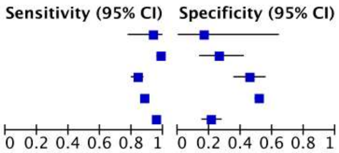

Combined Sensitivity = 0.93 (0.87-0.96); Inconsistency (I-square) = 94.9%
Combined Specificity = 0.33 (0.21-0.48); Inconsistency (I-square) = 94.0%

Moderate-to-severe OSA (AHI ≥ 15)

Study	TP	FP	FN	TN	Sensitivity (95% CI)	Specificity (95% CI)
Baldini 2017	218	92	2	15	0.99 [0.97, 1.00]	0.14 [0.08, 0.22]
Duarte 2017	210	142	27	77	0.89 [0.84, 0.92]	0.35 [0.29, 0.42]
Duarte 2020	3897	1963	334	1183	0.92 [0.91, 0.93]	0.38 [0.36, 0.39]
Saldias Penafiel 2019	838	509	20	97	0.98 [0.96, 0.99]	0.16 [0.13, 0.19]

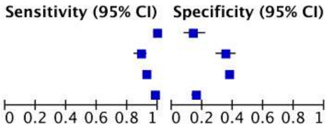

Combined Sensitivity = 0.96 (0.91-0.98); Inconsistency (I-square) = 95.6%
Combined Specificity = 0.25 (0.15-0.38); Inconsistency (I-square) = 97.8%

Severe OSA (AHI ≥ 30)

Study	TP	FP	FN	TN	Sensitivity (95% CI)	Specificity (95% CI)
Baldini 2017	161	149	0	17	1.00 [0.98, 1.00]	0.10 [0.06, 0.16]
Duarte 2017	124	228	6	98	0.95 [0.90, 0.98]	0.30 [0.25, 0.35]
Duarte 2020	2605	3254	107	1411	0.96 [0.95, 0.97]	0.30 [0.29, 0.32]

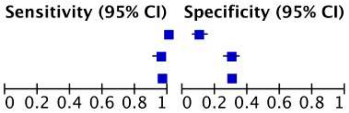

Combined Sensitivity = 0.96 (0.95-0.97); Inconsistency (I-square) = 79.5%
Combined Specificity = 0.30 (0.28-0.31); Inconsistency (I-square) = 92.0%

© 2021 Pivetta B et al. *JAMA Network Open.*

C. Europe

All OSA (AHI ≥ 5)

Study	TP	FP	FN	TN	Sensitivity (95% CI)	Specificity (95% CI)
Bille 2015	26	3	4	10	0.87 [0.69, 0.96]	0.77 [0.46, 0.95]
Christensson 2018	286	115	15	33	0.95 [0.92, 0.97]	0.22 [0.16, 0.30]
Cowan 2014	90	20	7	12	0.93 [0.86, 0.97]	0.38 [0.21, 0.56]
Korvel-Hanquist 2018	148	49	8	3	0.95 [0.90, 0.98]	0.06 [0.01, 0.16]
Kuczynski 2019	851	239	9	24	0.99 [0.98, 1.00]	0.09 [0.06, 0.13]
Pataka 2014	1441	305	57	50	0.96 [0.95, 0.97]	0.14 [0.11, 0.18]
Pataka 2019	459	80	149	12	0.75 [0.72, 0.79]	0.13 [0.07, 0.22]
Rebelo-Marques 2017	214	28	3	14	0.99 [0.96, 1.00]	0.33 [0.20, 0.50]
Reis 2015	157	24	11	23	0.93 [0.89, 0.97]	0.49 [0.34, 0.64]

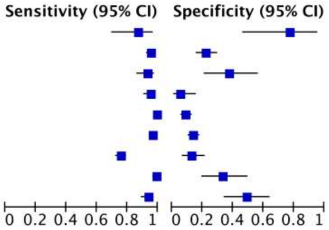

Combined Sensitivity = 0.95 (0.90-0.97); Inconsistency (I-square) = 99.0%
Combined Specificity = 0.24 (0.13-0.39); Inconsistency (I-square) = 92.1%

Moderate-to-severe OSA (AHI ≥ 15)

Study	TP	FP	FN	TN	Sensitivity (95% CI)	Specificity (95% CI)
Bille 2015	20	9	1	13	0.95 [0.76, 1.00]	0.59 [0.36, 0.79]
Christensson 2018	170	231	4	44	0.98 [0.94, 0.99]	0.16 [0.12, 0.21]
Cowan 2014	56	55	0	18	1.00 [0.94, 1.00]	0.25 [0.15, 0.36]
Korvel-Hanquist 2018	104	93	2	9	0.98 [0.93, 1.00]	0.09 [0.04, 0.16]
Kuczynski 2019	574	510	6	33	0.99 [0.98, 1.00]	0.06 [0.04, 0.08]
Pataka 2014	1221	213	26	393	0.98 [0.97, 0.99]	0.65 [0.61, 0.69]
Pataka 2019	459	146	70	25	0.87 [0.84, 0.90]	0.15 [0.10, 0.21]
Pataka 2020	294	131	98	177	0.75 [0.70, 0.79]	0.57 [0.52, 0.63]
Rebelo-Marques 2017	153	90	2	14	0.99 [0.95, 1.00]	0.13 [0.08, 0.22]
Reis 2015	113	68	5	29	0.96 [0.90, 0.99]	0.30 [0.21, 0.40]

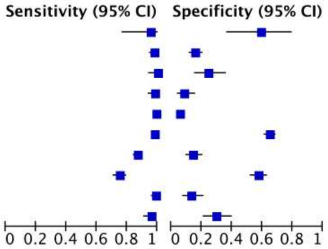

Combined Sensitivity = 0.97 (0.93-0.97); Inconsistency (I-square) = 97.7%
Combined Specificity = 0.25 (0.14-0.40); Inconsistency (I-square) = 98.8%

Severe OSA (AHI ≥ 30)

Study	TP	FP	FN	TN	Sensitivity (95% CI)	Specificity (95% CI)
Bille 2015	10	20	0	13	1.00 [0.69, 1.00]	0.39 [0.23, 0.58]
Korvel-Hanquist 2018	70	127	1	10	0.99 [0.92, 1.00]	0.07 [0.04, 0.13]
Pataka 2014	889	545	9	410	0.99 [0.98, 1.00]	0.43 [0.40, 0.46]
Pataka 2019	389	211	12	88	0.97 [0.95, 0.98]	0.29 [0.24, 0.35]
Rebelo-Marques 2017	80	163	0	16	1.00 [0.95, 1.00]	0.09 [0.05, 0.14]
Reis 2015	61	119	1	34	0.98 [0.91, 1.00]	0.22 [0.16, 0.30]

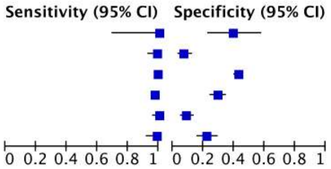

Combined Sensitivity = 0.99 (0.97-0.99); Inconsistency (I-square) = 43.4%
Combined Specificity = 0.22 (0.12-0.36); Inconsistency (I-square) = 98.1%

© 2021 Pivetta B et al. *JAMA Network Open.*

D. Middle East

All OSA (AHI ≥ 5)

Study	TP	FP	FN	TN	Sensitivity (95% CI)	Specificity (95% CI)
Acar 2013	70	34	5	1	0.93 [0.85, 0.98]	0.03 [0.00, 0.15]
Alhouqani 2015	148	20	16	9	0.90 [0.85, 0.94]	0.31 [0.15, 0.51]
Amra 2018	312	3	71	14	0.81 [0.77, 0.85]	0.82 [0.57, 0.96]
Arslan 2019	885	83	19	16	0.98 [0.97, 0.99]	0.16 [0.10, 0.25]
Avincsal 2017	116	29	14	3	0.89 [0.83, 0.94]	0.09 [0.02, 0.25]
BaHammam 2015	81	2	13	4	0.86 [0.78, 0.92]	0.67 [0.22, 0.96]
Bingol 2016	179	16	0	1	1.00 [0.98, 1.00]	0.06 [0.00, 0.29]
El-Sayed 2012	196	27	8	3	0.96 [0.92, 0.98]	0.10 [0.02, 0.27]
Kashaninasab 2017	161	5	82	2	0.66 [0.60, 0.72]	0.29 [0.04, 0.71]
Mergen 2019	204	1	10	2	0.95 [0.92, 0.98]	0.67 [0.09, 0.99]
Sadeghniiat-Haghighi 2015	401	101	38	63	0.91 [0.88, 0.94]	0.38 [0.31, 0.46]

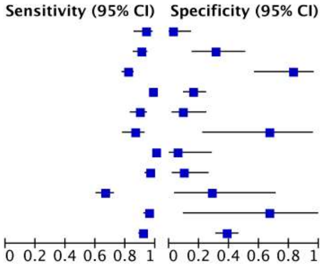

Combined Sensitivity = 0.93 (0.87-0.96); Inconsistency (I-square) = 96.9%
Combined Specificity = 0.24 (0.12-0.44); Inconsistency (I-square) = 90.2%

Moderate-to-severe OSA (AHI ≥ 15)

Study	TP	FP	FN	TN	Sensitivity (95% CI)	Specificity (95% CI)
Alhouqani 2015	119	49	4	21	0.97 [0.92, 0.99]	0.30 [0.20, 0.42]
Arslan 2019	665	303	10	25	0.99 [0.97, 0.99]	0.08 [0.05, 0.11]
Avincsal 2017	100	41	16	5	0.86 [0.79, 0.92]	0.11 [0.04, 0.24]
BaHammam 2015	81	5	4	10	0.95 [0.88, 0.99]	0.67 [0.38, 0.88]
El-Sayed 2012	173	50	4	7	0.98 [0.94, 0.99]	0.12 [0.05, 0.24]
Kashaninasab 2017	151	12	51	36	0.75 [0.68, 0.81]	0.75 [0.60, 0.86]
Sadeghniiat-Haghighi 2015	305	197	10	91	0.97 [0.94, 0.98]	0.32 [0.26, 0.37]

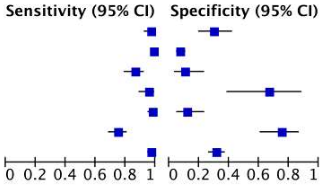

Combined Sensitivity = 0.95 (0.89-0.98); Inconsistency (I-square) = 97.3%
Combined Specificity = 0.28 (0.13-0.50); Inconsistency (I-square) = 96.4%

Severe OSA (AHI ≥ 30)

Study	TP	FP	FN	TN	Sensitivity (95% CI)	Specificity (95% CI)
Alhouqani 2015	85	83	2	23	0.98 [0.92, 1.00]	0.22 [0.14, 0.31]
Avincsal 2017	79	62	7	14	0.92 [0.84, 0.97]	0.18 [0.10, 0.29]
BaHammam 2015	45	9	18	28	0.71 [0.59, 0.82]	0.76 [0.59, 0.88]
El-Sayed 2012	146	77	2	9	0.99 [0.95, 1.00]	0.10 [0.05, 0.19]
Kashaninasab 2017	128	35	31	56	0.81 [0.73, 0.86]	0.62 [0.51, 0.72]
Sadeghniiat-Haghighi 2015	197	305	4	97	0.98 [0.95, 0.99]	0.24 [0.20, 0.29]

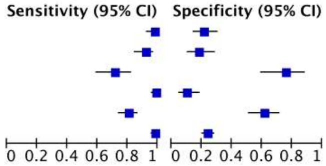

Combined Sensitivity = 0.94 (0.85-0.98); Inconsistency (I-square) = 93.9%
Combined Specificity = 0.33 (0.17-0.54); Inconsistency (I-square) = 95.6%

© 2021 Pivetta B et al. *JAMA Network Open.*

E. East Asia

All OSA (AHI ≥ 5)

Study	TP	FP	FN	TN	Sensitivity (95% CI)	Specificity (95% CI)
Byun 2020	539	126	30	83	0.95 [0.93, 0.96]	0.40 [0.33, 0.47]
Hu 2019	152	17	16	11	0.90 [0.85, 0.94]	0.39 [0.22, 0.59]
Luo 2014	186	8	10	8	0.95 [0.91, 0.98]	0.50 [0.25, 0.75]
Peng 2018	227	80	88	84	0.72 [0.67, 0.77]	0.51 [0.43, 0.59]

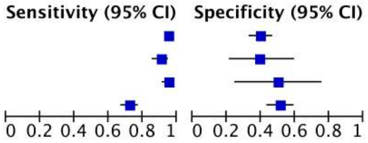

Combined Sensitivity = 0.90 (0.81-0.96); Inconsistency (I-square) = 97.5%
Combined Specificity = 0.44 (0.37-0.50); Inconsistency (I-square) = 59.3%

Moderate-to-severe OSA (AHI ≥ 15)

Study	TP	FP	FN	TN	Sensitivity (95% CI)	Specificity (95% CI)
Byun 2020	411	254	13	100	0.97 [0.95, 0.98]	0.28 [0.24, 0.33]
Hu 2019	131	38	9	18	0.94 [0.88, 0.97]	0.32 [0.20, 0.46]
Luo 2014	164	30	6	12	0.96 [0.92, 0.99]	0.29 [0.16, 0.45]
Peng 2018	152	155	50	122	0.75 [0.69, 0.81]	0.44 [0.38, 0.50]

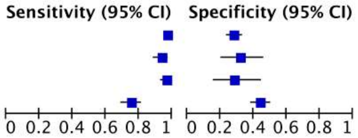

Combined Sensitivity = 0.93 (0.84-0.97); Inconsistency (I-square) = 96.8%
Combined Specificity = 0.33 (0.27-0.41); Inconsistency (I-square) = 83.4%

Severe OSA (AHI ≥ 30)

Study	TP	FP	FN	TN	Sensitivity (95% CI)	Specificity (95% CI)
Hu 2019	105	64	4	23	0.96 [0.91, 0.99]	0.26 [0.18, 0.37]
Luo 2014	125	69	3	15	0.98 [0.93, 1.00]	0.18 [0.10, 0.28]
Peng 2018	90	239	28	122	0.76 [0.68, 0.84]	0.34 [0.29, 0.39]

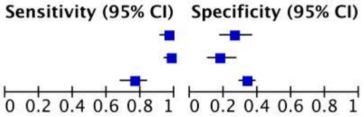

Combined Sensitivity = 0.90 (0.87-0.93); Inconsistency (I-square) = 94.5%
Combined Specificity = 0.30 (0.26-0.34); Inconsistency (I-square) = 78.9%

© 2021 Pivetta B et al. *JAMA Network Open.*

F. South/Southeast Asia

All OSA (AHI ≥ 5)

Study	TP	FP	FN	TN	Sensitivity (95% CI)	Specificity (95% CI)
Abdullah 2018	81	0	53	0	0.60 [0.52, 0.69]	Not estimable
Banhiran 2014	194	42	28	39	0.87 [0.82, 0.91]	0.48 [0.37, 0.60]
Chakrabarti 2019	51	0	24	5	0.68 [0.56, 0.78]	1.00 [0.48, 1.00]
Loh 2018	269	40	159	123	0.63 [0.58, 0.67]	0.75 [0.68, 0.82]
Ong 2010	207	34	37	41	0.85 [0.80, 0.89]	0.55 [0.43, 0.66]
Perumalsamy 2017	54	8	0	0	1.00 [0.93, 1.00]	0.00 [0.00, 0.37]
Vulli 2019	29	2	1	3	0.97 [0.83, 1.00]	0.60 [0.15, 0.95]

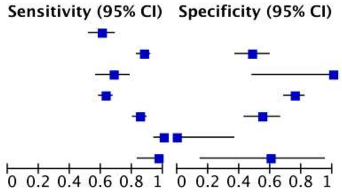

Combined Sensitivity = 0.75 (0.73-0.78); Inconsistency (I-square) = 95.1%
Combined Specificity = 0.60 (0.46-0.73); Inconsistency (I-square) = 84.7%

Moderate-to-severe OSA (AHI ≥ 15)

Study	TP	FP	FN	TN	Sensitivity (95% CI)	Specificity (95% CI)
Abdullah 2018	58	11	38	27	0.60 [0.50, 0.70]	0.71 [0.54, 0.85]
Banhiran 2014	138	98	11	56	0.93 [0.87, 0.96]	0.36 [0.29, 0.44]
Ong 2010	151	90	15	63	0.91 [0.86, 0.95]	0.41 [0.33, 0.49]
Vulli 2019	28	3	0	4	1.00 [0.88, 1.00]	0.57 [0.18, 0.90]

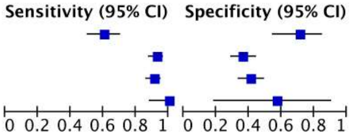

Combined Sensitivity = 0.89 (0.73-0.96); Inconsistency (I-square) = 95.6%
Combined Specificity = 0.45 (0.30-0.61); Inconsistency (I-square) = 81.0%

Severe OSA (AHI ≥ 30)

Study	TP	FP	FN	TN	Sensitivity (95% CI)	Specificity (95% CI)
Banhiran 2014	94	142	3	64	0.97 [0.91, 0.99]	0.31 [0.25, 0.38]
Ong 2010	108	133	5	73	0.96 [0.90, 0.99]	0.35 [0.29, 0.42]
Vulli 2019	13	18	0	4	1.00 [0.75, 1.00]	0.18 [0.05, 0.40]

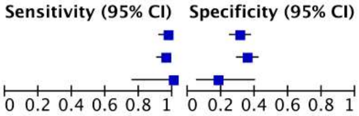

Combined Sensitivity = 0.96 (0.93-0.98); Inconsistency (I-square) = 0.0%
Combined Specificity = 0.33 (0.28-0.37); Inconsistency (I-square) = 38.9%

AHI: Apnea-hypopnea index; OSA: Obstructive sleep apnea; CI: Confidence interval; FN: False negative; FP: False positive; TN: True negative; TP: True positive.

© 2021 Pivetta B et al. *JAMA Network Open.*

eTable 1. Search Strategy

Search history record for Systematic Review: STOP-BANG Questionnaire Screening Tool

Review/Search Topic: STOP-BANG Questionnaire Screening Tool; years = >2008; no other limits applied	Searcher: Marina Englesakis

Databases	Database Dates covered	Date Database was searched	# Citations	# Duplicate Citations	Total Citations remaining	Notes/Comments
Medline/MedlineALL (Ovid)	1946 – March 26, 2020	March 30, 2020	396			
Embase (Ovid)	1947 – March 27, 2020	March 30, 2020	763			
EmCare Nursing (Ovid)	1995 – present	March 30, 2020	9			
Cochrane Central Register of Controlled Trials (Ovid)	1991 – present	March 30, 2020	37			
Cochrane Database of Systematic Reviews (Ovid)	2005 – present	March 30, 2020	0			
APA PsycINFO (Ovid)	1806 – March Week #4, 2020	March 30, 2020	100			
Journals@Ovid via University of Toronto (full-text searching	March 30 2020	March 30, 2020	689			
Web of Science (Clarivate) including citation search (Chung, Anesthesiology 2008, 812)	1900 – March 27, 2020	March 30, 2020	1127			
Scopus (Elsevier)	1960 – present	March 30, 2020	474			
CINAHL with Full Text (EbscoHost)	1982 – present	March 30, 2020	276			

© 2021 Pivetta B et al. *JAMA Network Open.*

		Totals:	3871	0	0	Results in a compressed EndNote Library

© 2021 Pivetta B et al. *JAMA Network Open.*

eTable 2. Excluded Studies and Reasons for Exclusion

Study ID	Study title	Population	Reasons for exclusion
**Ha 2014**	Evaluation and validation of four translated Chinese questionnaires for obstructive sleep apnea patients in Hong Kong	Chinese 141	Modified STOP-Bang questionnaire: BMI > 30
**Doshi 2015**	STOP-BANG questionnaire as a screening tool for diagnosis of obstructive sleep apnea by unattended portable monitoring sleep study	American 502	No data provided for any OSA prevalence
**Kim 2015**	The utility of three screening questionnaires for obstructive sleep apnea in a sleep clinic setting.	Korean 592	Modified STOP-Bang questionnaire: BMI > 30
**Ji 2017**	STOP-Bang Questionnaire in Patients with Rapid Eye Movement Sleep Behavior Disorder	Korean 65	Included patients with other sleep disorders, i.e. rapid eye movement sleep behavior disorder
**Prasad 2017**	Assessing the likelihood of obstructive sleep apnea: a comparison of nine screening questionnaires	Indian 210	Modified STOP-Bang questionnaire: BMI > 25
**Suliman 2017**	Value of STOP-BANG and Berlin questionnaires in the diagnosis and severity prediction of obstructive sleep apnea hypopnea syndrome	Egyptian 50	Provided no data related to STOP-Bang score of 3
**Hong 2018**	Validation of the NoSAS Score for the Screening of Sleep-Disordered Breathing: A Hospital-Based Retrospective Study in China	Chinese 2208	Modified AHI cut-off: AHI ≥ 20
**Kim 2019**	Real-world STOPBANG: how useful is STOPBANG for sleep clinics?	Korean 541	Provided no data related to STOP-Bang score of 3
**Ozturk 2019**	Diagnostic Accuracy of a Modified STOP-BANG Questionnaire with National Anthropometric Obesity Indexes	Turkish 185	Modified STOP-Bang questionnaire: neck circumference (40 cm, 36 cm)
**Sweed 2019**	Validation of the NoSAS score for the screening of sleep-disordered breathing: a retrospective study in Egypt	Egyptian 362	Modified AHI cut-off: AHI ≥ 20
**Rong 2020**	Validation of the NoSAS Score for the Screening of Sleep-Disordered Breathing in a Sleep Clinic	Chinese 596	Modified STOP-Bang questionnaire: BMI > 30

AHI: Apnea-hypopnea index; BMI: Body mass index; OSA: Obstructive sleep apnea.

© 2021 Pivetta B et al. *JAMA Network Open.*

eTable 3. Characteristics of Included Studies

Study ID	Prevalence of OSA n (%)	OSA definition	No OSA AHI <5 n (%)	Mild OSA AHI ≥5 to <15 n (%)	Moderate OSA AHI ≥15 to <30 or RDI >15 to <30 n (%)	Severe OSA AHI ≥ 30 or RDI ≥ 30 n (%)
North America						
Boynton 2013	169 (77)	AHI > 5	50 (23)	66 (30)	41 (19)	62 (28)
Farney 2011	1,274 (90)	AHI ≥ 5	150 (11)	315 (22)	379 (27)	580 (40)
McMahon 2017	216 (64)	AHI ≥ 5	122 (36)	130 (38)	53 (16)	33 (10)
Miller 2018	108 (76)	AHI ≥ 15	34 (24)	43 (30)	34 (24)	31 (22)
Mou 2019	727 (78)	AHI ≥ 5	208 (22)	258 (28)	189 (20)	280 (30)
Orbea 2020	42 (64)	AHI ≥ 5	24 (36)	28 (43)	8 (12)	6 (9)
Pereira 2013	116 (91)	AHI ≥ 5	12 (9)	28 (22)	32 (25)	56 (44)
Sangkum 2017	162 (78)	AHI > 5	46 (22)	62 (30)	40 (19)	60 (29)
Vana 2013	32 (68)	AHI ≥ 5	15 (32)	13 (28)	10 (21)	9 (19)
South America						
Andrade 2020	29 (83)	AHI ≥ 5	6 (17)	6 (17)	3 (9)	20 (57)
Baldini 2017	285 (87)	RDI ≥ 5	42 (13)	65 (20)	59 (18)	161 (49)
Duarte 2017	357 (78)	AHI ≥ 5	99 (22)	120 (26)	107 (23)	130 (29)
Duarte 2020	5,826 (79)	AHI ≥ 5	1551 (21)	1595 (22)	1519 (21)	2712 (36)
Saldias Penafiel 2018	880 (84)	AHI ≥ 5	170 (16)	271 (26)	231 (22)	378 (36)
Saldias Penafiel 2019	1,211 (83)	AHI ≥ 5	253 (17)	353 (24)	320 (22)	538 (37)
Europe						
Bille 2015	30 (70)	AHI > 5	13 (30)	9 (21)	11 (26)	10 (23)

© 2021 Pivetta B et al. *JAMA Network Open.*

Christensson 2018	301 (67)	AHI ≥ 5	148 (33)	127 (28)	174 (39)	NR
Cowan 2014	97 (75)	AHI ≥ 5	32 (25)	41 (32)	56 (43)	NR
Kørvel-Hanquist 2018	156 (75)	AHI ≥ 5	52 (25)	50 (24)	35 (17)	71 (34)
Kuczynski 2019	860 (77)	AHI ≥ 5	263 (23)	280 (25)	185 (17)	395 (35)
Pataka 2014	1,498 (81)	AHI ≥ 5	355 (19)	251 (14)	349 (19)	898 (48)
Pataka 2019	608 (87)	AHI ≥ 5	92 (13)	79 (11)	128 (18)	401 (58)
Pataka 2020	504 (72)	AHI ≥ 5	196 (28)	112 (16)	147 (21)	245 (35)
Rebelo-Marques 2017	219 (84)	AHI ≥ 5	42 (16)	64 (24)	75 (29)	80 (31)
Reis 2015	168 (78)	AHI > 5	47 (22)	50 (23)	56 (26)	62 (29)
Middle East						
Acar 2013	75 (68)	AHI > 5	35 (32)	75 (68)	NR	NR
Alhouqani 2015	164 (84)	AHI ≥ 5	29 (15)	41 (21)	36 (19)	87 (45)
Amra 2018	383 (96)	AHI ≥ 5	17 (4)	56 (14)	84 (21)	243 (61)
Arslan 2019	904 (90)	AHI ≥ 5	99 (10)	229 (23)	256 (26)	419 (42)
Avincsal 2017	130 (80)	AHI > 5	32 (20)	14 (9)	30 (19)	86 (53)
BaHammam 2015	94 (94)	AHI ≥ 5	6 (6)	9 (9)	22 (22)	63 (63)
Bingol 2016	179 (91)	AHI ≥ 5	17 (9)	179 (91)	NR	NR
El-Sayed 2012	204 (87)	AHI ≥ 5	30 (13)	27 (12)	29 (12)	148 (63)
Kashaninasab 2017	243 (97)	AHI > 5	7 (3)	41 (16)	43 (17)	159 (64)
Mergen 2019	214 (99)	AHI > 5	3 (1)	25 (12)	45 (21)	144 (66)
Sadeghniiat-Haghighi 2015	439 (73)	AHI ≥ 5	164 (27)	124 (21)	114 (19)	201 (33)
East Asia						
Byun 2020	569 (73)	AHI > 5	209 (27)	145 (19)	127 (16)	297 (38)

© 2021 Pivetta B et al. *JAMA Network Open.*

Hu 2019	168 (86)	AHI ≥ 5	28 (14)	28 (14)	31 (16)	109 (56)
Luo 2014	196 (93)	AHI ≥ 5	16 (8)	26 (12)	42 (20)	128 (60)
Peng 2018	315 (66)	AHI ≥ 5	164 (34)	113 (24)	84 (18)	118 (25)
South/Southeast Asia						
Abdullah 2018	134 (100)	AHI ≥ 15	0 (0)	38 (28)	45 (34)	51 (38)
Banhiran 2014	222 (73)	AHI ≥ 5	81 (27)	73 (24)	52 (17)	97 (32)
Chakrabarti 2019	75 (94)	AHI ≥ 5	5 (6)	75 (94)	NR	NR
Loh 2018	428 (72)	AHI ≥ 5	163 (28)	155 (26)	91 (15)	182 (31)
Ong 2010	244 (77)	AHI > 5	75 (24)	78 (24)	53 (17)	113 (35)
Perumalsamy 2017	54 (87)	AHI ≥ 5	8 (13)	15 (24)	13 (21)	26 (42)
Vulli 2019	30 (86)	AHI ≥ 5	5 (14)	2 (6)	15 (43)	13 (37)

AHI: Apnea-hypopnea index; RDI: Respiratory disturbance index; NR: Not reported; OSA: Obstructive sleep apnea; PSG: Polysomnography; HSAT: Home sleep apnea testing; USA: United States of America.

© 2021 Pivetta B et al. *JAMA Network Open.*

eTable 4. Appraisal of Included Studies Based on Criteria for Internal Validity

Internal criteria	Valid reference standard	Definition of the disease based on reference standard	Blind execution of index test and reference test	Index test interpreted independently of clinical information	Study design
**Definition**	**Laboratory PSG (Lab PSG) or Portable PSG (Port PSG)**	**OSA diagnosed based on the PSG results (F)**	**PSG readings blinded to the questionnaire results and vice versa (F)**	**The questionnaire interpreted independently of clinical information (F)**	**Prospective or retrospective**
**North America**					
Farney 2011	Lab PSG	F	U	U	Retrospective
Boynton 2013	Lab PSG	F	F	F	Prospective
Pereira 2013	Lab/Port PSG	F	F	F	Prospective
Vana 2013	Lab PSG	F	U	U	Prospective
McMahon 2017	Lab PSG	F	U	U	Retrospective
Sangkum 2017	Lab PSG	F	F	U	Prospective
Miller 2018	Lab/Port PSG	F	U	U	Prospective
Mou 2019	Lab PSG	F	U	U	Prospective
Orbea 2020	Lab/Port PSG	F	U	U	Retrospective
**South America**					
Baldini 2017	Lab PSG	F	U	U	Retrospective
Duarte 2017	Lab PSG	F	F	F	Prospective
Saldias Penafiel 2018	Port PSG	F	F	F	Prospective
Saldias Penafiel 2019	Lab PSG	F	F	F	Prospective
Andrade 2020	Lab PSG	F	U	U	Prospective
Duarte 2020	Lab PSG	F	F	F	Prospective
**Europe**					
Cowan 2014	Port PSG	F	F	F	Prospective
Pataka 2014	Lab PSG	F	U	U	Retrospective
Bille 2015	Port PSG	F	U	U	Prospective
Reis 2015	Lab/Port PSG	F	F	F	Prospective
Rebelo-Marques 2017	Lab/Port PSG	F	F	F	Prospective
Christensson 2018	Port PSG	F	F	F	Prospective
Kørvel-Hanquist 2018	Port PSG	F	F	F	Prospective
Kuczynski 2019	Lab PSG	F	U	U	Retrospective
Pataka 2019	Lab PSG	F	U	U	Retrospective
Pataka 2020	Lab PSG	F	U	U	Prospective

© 2021 Pivetta B et al. *JAMA Network Open.*

**Middle East**					
El-Sayed 2012	Lab PSG	F	F	F	Prospective
Acar 2013	Lab PSG	F	U	U	Prospective
Alhouqani 2015	Lab PSG	F	U	U	Prospective
BaHammam 2015	Lab PSG	F	F	F	Prospective
Sadeghniiat-Haghighi 2015	Lab PSG	F	U	U	Prospective
Bingol 2016	Lab PSG	F	U	U	Prospective
Avincsal 2017	Lab PSG	F	F	F	Retrospective
Kashaninasab 2017	Lab PSG	F	U	U	Prospective
Amra 2018	Lab PSG	F	F	F	Prospective
Arslan 2019	Lab PSG	F	U	U	Prospective
Mergen 2019	Lab PSG	F	U	U	Retrospective
**East Asia**					
Luo 2014	Lab PSG	F	U	U	Prospective
Peng 2018	Lab PSG	F	U	U	Retrospective
Hu 2019	Lab PSG	F	F	F	Prospective
Byun 2020	Lab PSG	F	U	U	Prospective
**South/Southeast Asia**					
Ong 2010	Lab PSG	F	F	F	Prospective
Banhiran 2014	Lab PSG	F	F	F	Prospective
Perumalsamy 2017	Lab PSG	F	F	F	Prospective
Abdullah 2018	Lab PSG	F	U	U	Prospective
Loh 2018	Lab PSG	F	U	U	Retrospective
Chakrabarti 2019	Lab PSG	F	F	F	Retrospective
Vulli 2019	Lab PSG	F	U	U	Prospective

F: fully meeting criteria; P: partially meeting criteria; U: unsure/unclear if meeting criteria; N: not meeting criteria.

OSA: Obstructive sleep apnea; PSG: Polysomnography.

© 2021 Pivetta B et al. *JAMA Network Open.*

eTable 5. Appraisal of Included Studies Based on Criteria for External Validity

External criteria	Spectrum of diseases	Settings	Previous screening	Demographic information	Explication of cut-off point of index test	Percentage missing	Missing data management	Subject selection for reference test
Definition	Inclusion and exclusion criteria mentioned (F)	Enough information to identify setting (F)	No pre-screening before questionnaire application (F)	Age, gender, BMI data provided (F)	Results presented for AHI ≥ 5 or RDI ≥ 15 (F)	Percentage missing mentioned (F)	Analysis of missing data for basic characteristics (F)	All subjects were invited or randomly selected to do PSG (F)
**North America**								
Farney 2011	F	F	F	F	F	F	F	F
Boynton 2013	F	F	F	F	F	F	N	F
Pereira 2013	F	F	F	F	F	U	U	F
Vana 2013	F	F	F	F	F	F	N	F
McMahon 2017	F	F	F	F	F	F	P	F
Sangkum 2017	F	F	F	F	F	N	N	F
Miller 2018	F	F	F	P	F	F	U	F
Mou 2019	F	F	F	F	F	N	N	F
Orbea 2020	F	F	F	F	F	N/A	N/A	F
**South America**								
Baldini 2017	F	F	F	F	F	U	U	F
Duarte 2017	F	F	F	F	F	F	F	F
Saldias Penafiel 2018	F	F	F	F	F	N	N	F
Saldias Penafiel 2019	F	F	F	F	F	N	N	F
Andrade 2020	F	F	F	F	F	U	U	F
Duarte 2020	F	F	F	F	F	F	P	F
**Europe**								
Cowan 2014	F	F	F	F	F	F	F	F
Pataka 2014	F	F	F	F	F	F	U	F
Bille 2015	F	F	F	F	F	U	U	F
Reis 2015	F	F	F	F	F	F	P	F
Rebelo-Marques 2017	F	F	F	F	F	N/A	N/A	F
Christensson 2018	F	F	F	F	F	F	F	F
Kørvel-Hanquist 2018	F	F	F	F	F	N	N	F

© 2021 Pivetta B et al. *JAMA Network Open.*

Kuczynski 2019	F	F	F	F	F	F	P	F
Pataka 2019	F	F	F	F	F	F	N	F
Pataka 2020	F	F	F	F	F	U	U	F
**Middle East**								
El-Sayed 2012	F	F	F	F	F	N	N	F
Acar 2013	F	F	F	F	F	N	N	F
Alhouqani 2015	F	F	F	F	F	F	U	F
BaHammam 2015	F	F	F	F	F	N/A	N/A	
Sadeghniiat-Haghighi 2015	F	F	F	F	F	F	P	F
Bingol 2016	F	F	F	F	F	F	N	F
Avincsal 2017	F	F	F	F	F	F	F	F
Kashaninasab 2017	F	F	F	F	F	N/A	N/A	F
Amra 2018	F	F	F	F	F	N	N	F
Arslan 2019	F	F	F	F	F	U	U	F
Mergen 2019	F	F	F	F	F	F	N	F
**East Asia**								
Luo 2014	F	F	F	F	F	F	P	F
Peng 2018	F	F	F	F	F	F	P	F
Hu 2019	F	F	F	F	F	F	P	F
Byun 2020	F	F	F	F	F	F	U	F
**South/Southeast Asia**								
Ong 2010	F	F	F	F	F	F	F	F
Banhiran 2014	F	F	F	F	F	N/A	N/A	F
Perumalsamy 2017	F	F	F	P	F	N	N	F
Abdullah 2018	F	F	F	P	F	N	N	F
Loh 2018	F	F	F	F	F	F	P	F
Chakrabarti 2019	F	F	F	F	F	U	U	F
Vulli 2019	F	F	F	F	F	U	U	F

F: fully meeting criteria; P: partially meeting criteria; U: unsure/unclear if meeting criteria; N: not meeting criteria; N/A: not applicable.

AHI: Apnea-hypopnea index; BMI: Body mass index; RDI: Respiratory disturbance index.

© 2021 Pivetta B et al. *JAMA Network Open.*

eTable 6. Tables Describing the 2 × 2 Contingency Values and Test Characteristics of Individual Studies for All OSA (AHI ≥5), Moderate to Severe OSA (AHI ≥15), and Severe OSA (AHI ≥30)

Description of 2X2 contingency table:

	Polysomnography Positive (PSG +)	Polysomnography Negative (PSG -)	Total
STOP-Bang Positive (SB+)	True Positive	False Positive	
STOP-Bang Negative (SB-)	False Negative	True Negative	
Total			

2X2 contingency table for sleep clinic setting – All OSA (AHI≥ 5)

Study ID	TP	FP	FN	TN	Sensitivity (95% CI)	Specificity (95% CI)
**North America**						
Boynton 2013	139	26	30	24	0.82 [0.76, 0.88]	0.48 [0.34, 0.63]
Farney 2011	1140	63	134	87	0.89 [0.88, 0.91]	0.58 [0.50, 0.66]
McMahon 2017	181	100	35	22	0.84 [0.78, 0.88]	0.18 [0.12, 0.26]
Miller 2018	102	27	6	7	0.94 [0.88, 0.98]	0.21 [0.09, 0.38]
Mou 2019	676	143	51	65	0.93 [0.91, 0.95]	0.31 [0.25, 0.38]
Orbea 2020	28	12	14	12	0.67 [0.50, 0.80]	0.50 [0.29, 0.71]
Pereira 2013	104	7	12	5	0.90 [0.83, 0.95]	0.42 [0.15, 0.72]
Sangkum 2017	156	37	6	9	0.96 [0.92, 0.99]	0.20 [0.09, 0.34]
Vana 2013	30	10	2	5	0.94 [0.79, 0.99]	0.33 [0.12, 0.62]
**South America**						
Andrade 2020	27	5	2	1	0.93 [0.77, 0.99]	0.17 [0.00, 0.64]
Baldini 2017	279	31	6	11	0.98 [0.95, 0.99]	0.26 [0.14, 0.42]
Duarte 2017	298	54	59	45	0.83 [0.79, 0.87]	0.45 [0.35, 0.56]
Duarte 2020	5103	755	723	796	0.88 [0.87, 0.88]	0.51 [0.49, 0.54]
Saldias Penafiel 2018	836	134	44	36	0.95 [0.93, 0.96]	0.21 [0.15, 0.28]
**Europe**						
Bille 2015	26	3	4	10	0.87 [0.69, 0.96]	0.77 [0.46, 0.95]
Christensson 2018	286	115	15	33	0.95 [0.92, 0.97]	0.22 [0.16, 0.30]
Cowan 2014	90	20	7	12	0.93 [0.86, 0.97]	0.38 [0.21, 0.56]
Korvel-Hanquist 2018	148	49	8	3	0.95 [0.90, 0.98]	0.06 [0.01, 0.16]
Kuczynski 2019	851	239	9	24	0.99 [0.98, 1.00]	0.09 [0.06, 0.13]
Pataka 2014	1441	305	57	50	0.96 [0.95, 0.97]	0.14 [0.11, 0.18]
Pataka 2019	459	80	149	12	0.75 [0.72, 0.79]	0.13 [0.07, 0.22]
Rebelo-Marques 2017	214	28	3	14	0.99 [0.96, 1.00]	0.33 [0.20, 0.50]
Reis 2015	157	24	11	23	0.93 [0.89, 0.97]	0.49 [0.34, 0.64]
**Middle East**						

© 2021 Pivetta B et al. *JAMA Network Open.*

Acar 2013	70	34	5	1	0.93 [0.85, 0.98]	0.03 [0.00, 0.15]
Alhouqani 2015	148	20	16	9	0.90 [0.85, 0.94]	0.31 [0.15, 0.51]
Amra 2018	312	3	71	14	0.81 [0.77, 0.85]	0.82 [0.57, 0.96]
Arslan 2019	885	83	19	16	0.98 [0.97, 0.99]	0.16 [0.10, 0.25]
Avincsal 2017	116	29	14	3	0.89 [0.83, 0.94]	0.09 [0.02, 0.25]
BaHammam 2015	81	2	13	4	0.86 [0.78, 0.92]	0.67 [0.22, 0.96]
Bingol 2016	179	16	0	1	1.00 [0.98, 1.00]	0.06 [0.00, 0.29]
El-Sayed 2012	196	27	8	3	0.96 [0.92, 0.98]	0.10 [0.02, 0.27]
Kashaninasab 2017	161	5	82	2	0.66 [0.60, 0.72]	0.29 [0.04, 0.71]
Mergen 2019	204	1	10	2	0.95 [0.92, 0.98]	0.67 [0.09, 0.99]
Sadeghniiat-Haghighi 2015	401	101	38	63	0.91 [0.88, 0.94]	0.38 [0.31, 0.46]
**East Asia**						
Byun 2020	539	126	30	83	0.95 [0.93, 0.96]	0.40 [0.33, 0.47]
Hu 2019	152	17	16	11	0.90 [0.85, 0.94]	0.39 [0.22, 0.59]
Luo 2014	186	8	10	8	0.95 [0.91, 0.98]	0.50 [0.25, 0.75]
Peng 2018	227	80	88	84	0.72 [0.67, 0.77]	0.51 [0.43, 0.59]
**South/Southeast Asia**						
Abdullah 2018	81	0	53	0	0.60 [0.52, 0.69]	Not estimable
Banhiran 2014	194	42	28	39	0.87 [0.82, 0.91]	0.48 [0.37, 0.60]
Chakrabarti 2019	51	0	24	5	0.68 [0.56, 0.78]	1.00 [0.48, 1.00]
Loh 2018	269	40	159	123	0.63 [0.58, 0.67]	0.75 [0.68, 0.82]
Ong 2010	207	34	37	41	0.85 [0.80, 0.89]	0.55 [0.43, 0.66]
Perumalsamy 2017	54	8	0	0	1.00 [0.93, 1.00]	0.00 [0.00, 0.37]
Vulli 2019	29	2	1	3	0.97 [0.83, 1.00]	0.60 [0.15, 0.95]

FN: False negative; FP: False positive; TN: True negative; TP: True positive; CI: confidence interval.

© 2021 Pivetta B et al. *JAMA Network Open.*

2X2 contingency table for sleep clinic setting – Moderate-to-severe OSA (AHI ≥ 15)

Study ID	TP	FP	FN	TN	Sensitivity (95% CI)	Specificity (95% CI)
**North America**						
Boynton 2013	96	69	7	47	0.93 [0.86, 0.97]	0.41 [0.32, 0.50]
Farney 2011	895	307	64	158	0.93 [0.92, 0.95]	0.34 [0.30, 0.38]
McMahon 2017	77	204	9	48	0.90 [0.81, 0.95]	0.19 [0.14, 0.24]
Miller 2018	62	67	3	10	0.95 [0.87, 0.99]	0.13 [0.06, 0.23]
Mou 2019	447	372	22	94	0.95 [0.93, 0.97]	0.20 [0.17, 0.24]
Orbea 2020	11	29	3	23	0.79 [0.49, 0.95]	0.44 [0.30, 0.59]
Pereira 2013	82	29	6	11	0.93 [0.86, 0.97]	0.28 [0.15, 0.44]
Sangkum 2017	98	95	2	13	0.98 [0.93, 1.00]	0.12 [0.07, 0.20]
Vana 2013	18	22	1	6	0.95 [0.74, 1.00]	0.21 [0.08, 0.41]
**South America**						
Baldini 2017	218	92	2	15	0.99 [0.97, 1.00]	0.14 [0.08, 0.22]
Duarte 2017	210	142	27	77	0.89 [0.84, 0.92]	0.35 [0.29, 0.42]
Duarte 2020	3897	1963	334	1183	0.92 [0.91, 0.93]	0.38 [0.36, 0.39]
Saldias Penafiel 2019	838	509	20	97	0.98 [0.96, 0.99]	0.16 [0.13, 0.19]
**Europe**						
Bille 2015	20	9	1	13	0.95 [0.76, 1.00]	0.59 [0.36, 0.79]
Christensson 2018	170	231	4	44	0.98 [0.94, 0.99]	0.16 [0.12, 0.21]
Cowan 2014	56	55	0	18	1.00 [0.94, 1.00]	0.25 [0.15, 0.36]
Korvel-Hanquist 2018	104	93	2	9	0.98 [0.93, 1.00]	0.09 [0.04, 0.16]
Kuczynski 2019	574	510	6	33	0.99 [0.98, 1.00]	0.06 [0.04, 0.08]
Pataka 2014	1221	213	26	393	0.98 [0.97, 0.99]	0.65 [0.61, 0.69]
Pataka 2019	459	146	70	25	0.87 [0.84, 0.90]	0.15 [0.10, 0.21]
Pataka 2020	294	131	98	177	0.75 [0.70, 0.79]	0.57 [0.52, 0.63]
Rebelo-Marques 2017	153	90	2	14	0.99 [0.95, 1.00]	0.13 [0.08, 0.22]
Reis 2015	113	68	5	29	0.96 [0.90, 0.99]	0.30 [0.21, 0.40]
**Middle East**						
Alhouqani 2015	119	49	4	21	0.97 [0.92, 0.99]	0.30 [0.20, 0.42]
Arslan 2019	665	303	10	25	0.99 [0.97, 0.99]	0.08 [0.05, 0.11]
Avincsal 2017	100	41	16	5	0.86 [0.79, 0.92]	0.11 [0.04, 0.24]
BaHammam 2015	81	5	4	10	0.95 [0.88, 0.99]	0.67 [0.38, 0.88]
El-Sayed 2012	173	50	4	7	0.98 [0.94, 0.99]	0.12 [0.05, 0.24]
Kashaninasab 2017	151	12	51	36	0.75 [0.68, 0.81]	0.75 [0.60, 0.86]
Sadeghniiat-Haghighi 2015	305	197	10	91	0.97 [0.94, 0.98]	0.32 [0.26, 0.37]
**East Asia**						
Byun 2020	411	254	13	100	0.97 [0.95, 0.98]	0.28 [0.24, 0.33]
Hu 2019	131	38	9	18	0.94 [0.88, 0.97]	0.32 [0.20, 0.46]
Luo 2014	164	30	6	12	0.96 [0.92, 0.99]	0.29 [0.16, 0.45]
Peng 2018	152	155	50	122	0.75 [0.69, 0.81]	0.44 [0.38, 0.50]

© 2021 Pivetta B et al. *JAMA Network Open.*

**South/Southeast Asia**						
Abdullah 2018	58	11	38	27	0.60 [0.50, 0.70]	0.71 [0.54, 0.85]
Banhiran 2014	138	98	11	56	0.93 [0.87, 0.96]	0.36 [0.29, 0.44]
Ong 2010	151	90	15	63	0.91 [0.86, 0.95]	0.41 [0.33, 0.49]
Vulli 2019	28	3	0	4	1.00 [0.88, 1.00]	0.57 [0.18, 0.90]

FN: False negative; FP: False positive; TN: True negative; TP: True positive, CI: confidence interval.

© 2021 Pivetta B et al. *JAMA Network Open.*

2X2 contingency table for sleep clinic setting – Severe OSA (AHI ≥ 30)

Study ID	TP	FP	FN	TN	Sensitivity (95% CI)	Specificity (95% CI)
**North America**						
Boynton 2013	61	104	2	52	0.97 [0.89, 1.00]	0.33 [0.26, 0.41]
Farney 2011	555	647	25	197	0.96 [0.94, 0.97]	0.23 [0.21, 0.26]
McMahon 2017	29	252	4	53	0.88 [0.72, 0.97]	0.17 [0.13, 0.22]
Miller 2018	30	99	1	12	0.97 [0.83, 1.00]	0.11 [0.06, 0.18]
Mou 2019	270	549	10	106	0.96 [0.94, 0.98]	0.16 [0.13, 0.19]
Orbea 2020	4	35	2	25	0.67 [0.22, 0.96]	0.42 [0.29, 0.55]
Pereira 2013	54	57	2	15	0.96 [0.88, 1.00]	0.21 [0.12, 0.32]
Sangkum 2017	59	134	1	14	0.98 [0.91, 1.00]	0.09 [0.05, 0.15]
**South America**						
Baldini 2017	161	149	0	17	1.00 [0.98, 1.00]	0.10 [0.06, 0.16]
Duarte 2017	124	228	6	98	0.95 [0.90, 0.98]	0.30 [0.25, 0.35]
Duarte 2020	2605	3254	107	1411	0.96 [0.95, 0.97]	0.30 [0.29, 0.32]
**Europe**						
Bille 2015	10	20	0	13	1.00 [0.69, 1.00]	0.39 [0.23, 0.58]
Korvel-Hanquist 2018	70	127	1	10	0.99 [0.92, 1.00]	0.07 [0.04, 0.13]
Pataka 2014	889	545	9	410	0.99 [0.98, 1.00]	0.43 [0.40, 0.46]
Pataka 2019	389	211	12	88	0.97 [0.95, 0.98]	0.29 [0.24, 0.35]
Rebelo-Marques 2017	80	163	0	16	1.00 [0.95, 1.00]	0.09 [0.05, 0.14]
Reis 2015	61	119	1	34	0.98 [0.91, 1.00]	0.22 [0.16, 0.30]
**Middle East**						
Alhouqani 2015	85	83	2	23	0.98 [0.92, 1.00]	0.22 [0.14, 0.31]
Avincsal 2017	79	62	7	14	0.92 [0.84, 0.97]	0.18 [0.10, 0.29]
BaHammam 2015	45	9	18	28	0.71 [0.59, 0.82]	0.76 [0.59, 0.88]
El-Sayed 2012	146	77	2	9	0.99 [0.95, 1.00]	0.10 [0.05, 0.19]
Kashaninasab 2017	128	35	31	56	0.81 [0.73, 0.86]	0.62 [0.51, 0.72]
Sadeghniiat-Haghighi 2015	197	305	4	97	0.98 [0.95, 0.99]	0.24 [0.20, 0.29]
**East Asia**						
Hu 2019	105	64	4	23	0.96 [0.91, 0.99]	0.26 [0.18, 0.37]
Luo 2014	125	69	3	15	0.98 [0.93, 1.00]	0.18 [0.10, 0.28]
Peng 2018	90	239	28	122	0.76 [0.68, 0.84]	0.34 [0.29, 0.39]
**South/Southeast Asia**						
Banhiran 2014	94	142	3	64	0.97 [0.91, 0.99]	0.31 [0.25, 0.38]
Ong 2010	108	133	5	73	0.96 [0.90, 0.99]	0.35 [0.29, 0.42]
Vulli 2019	13	18	0	4	1.00 [0.75, 1.00]	0.18 [0.05, 0.40]

FN: False negative; FP: False positive; TN: True negative; TP: True positive, CI: confidence interval.

© 2021 Pivetta B et al. *JAMA Network Open.*

eTable 7. Specificity Analysis of the STOP-Bang Questionnaire for Various Subgroups According to the Severity of OSA

A. All OSA (Apnea-hypopnea index ≥ 5)

Specificity			
Co-variate (number of studies)	**Point Estimate (95% CI) Heterogeneity**	**Coefficient (SE)**	**p-value**
Age (42)	34.3 (24.7 – 45.4)[96]	0.049 (0.062)	0.434
Gender (45)	33.6 (24.5 – 44.1)[96]	-0.012 (0.014)	0.402
BMI (41)	34.4 (24.7 – 45.7)[96]	0.062 (0.084)	0.464
Neck circumference (34)	32.9 (22.3 – 45.6)[96]	0.148 (0.221)	0.502
Sample size			
> 200 (30)<br>< 200 (15)	29.3 (19.6 – 41.3)[97]<br>45.6 (28.2 – 64.2)[86]	-0.860 (0.506)	0.089
Study type			
Prospective (19)<br>Retrospective (12)<br>Cross sectional (14)	37.0 (20.9 – 56.8)[97]<br>25.9 (14.2 – 42.3)[94]<br>35.0 (21.0 – 52.2)[93]	0.947 (0.565)<br>-0.100 (0.613)	0.093<br>0.870
Validation tool			
Lab PSG (39)<br>HSAT (6)	34.7 (24.7 – 46.4)[96]<br>26.6 (13.6 – 45.5)[87]	-1.499 (0.815)	0.066
OSA criteria			
AHI ≥ 5 (44)<br>RDI ≥ 5 (1)	33.8 (24.5 – 44.5)[96]<br>26.2 (15.1 – 41.4)[-]	-1.210 (1.619)	0.455
Prevalence (45)	33.6 (24.5 – 44.1)[96]	-0.028 (0.027)	0.302
North America (9)<br>South America (5)<br>Europe (9)<br>Middle East (11)<br>East Asia (4)<br>South/Southeast Asia (7)	34.5 (16.5 – 58.5)[95]<br>57.5 (14.2 – 91.7)[98]<br>40.2 (21.3 – 62.6)[94]<br>16.4 (5.3 – 41.0)[94]<br>37.2 (23.7 – 53.2)[88]<br>33.4 (22.8 – 46.0)[75]	-0.031(0.736)<br>1.023 (0.925)<br>0.441 (0.778)<br>-1.390 (0.754)<br>0.285 (0.900)	0.967<br>0.268<br>0.570<br>0.065<br>0.752

© 2021 Pivetta B et al. *JAMA Network Open.*

B. Moderate -to-severe OSA (Apnea-hypopnea index ≥ 15)

Specificity			
**Co-variate (number of studies)**	**Point Estimate (95% CI)** ^{Heterogeneity}	**Coefficient (SE)**	**p-value**
Age (35)	28.2 (23.0 – 34.0)^{96}	-0.068 (0.061)	0.061
Gender (38)	28.2 (23.4 – 33.7)^{96}	-0.009 (0.013)	0.507
BMI (33)	27.3 (22.5 – 32.8)^{95}	-0.084 (0.042)	0.044
Neck circumference (27)	30.8 (25.5 – 36.7)^{95}	-0.007 (0.034)	0.830
Sample size			
> 200 (27)<br>< 200 (11)	25.7 (20.4 – 31.8)^{97}<br>36.3 (25.3 – 49.0)^{83}	-0.491 (0.373)	0.188
Study type			
Prospective (19)<br>Retrospective (9)<br>Cross sectional (10)	29.2 (22.3 – 37.3)^{93}<br>24.3 (13.2 – 40.4)^{98}<br>29.8 (21.5 – 39.7)^{95}	0.152 (0.380)<br>-0.097 (0.488)	0.689<br>0.842
Validation tool			
Lab PSG (33)<br>HSAT (5)	29.0 (23.7 – 34.8)^{96}<br>23.4 (13.3 – 37.9)^{86}	-0.575 (0.616)	0.350
OSA criteria			
AHI ≥ 5 (37)<br>RDI ≥ 5 (1)	28.7 (23.7 – 34.3)^{96}<br>14.0 (8.00 – 22.0)$^{-}$	-0.770 (1.049)	0.463
Prevalence (38)	28.2 (23.4 – 33.7)^{96}	0.007 (0.016)	0.681
North America (9)<br>South America (4)<br>Europe (10)<br>Middle East (7)<br>East Asia (4)<br>South/Southeast Asia (4)	24.7 (18.4 – 32.3)^{88}<br>24.5 (14.3 – 38.8)^{97}<br>24.9 (12.5 – 43.5)^{98}<br>28.2 (14.0 – 48.8)^{94}<br>33.6 (24.6 – 43.9)^{83}<br>48.5 (34.8 – 62.4)^{79}	-1.286 (0.619)<br>-0.809 (0.671)<br>-0.859 (0.630)<br>-0.862 (0.546)<br>-0.593 (0.635)	0.038<br>0.228<br>0.173<br>0.115<br>0.350

© 2021 Pivetta B et al. *JAMA Network Open.*

C. Severe OSA (Apnea-hypopnea index ≥ 30)

Specificity			
Co-variate (number of studies)	**Point Estimate (95% CI)** Heterogeneity	**Coefficient (SE)**	**p-value**
Age (26)	24.7 (21.1 – 28.7)[93]	-0.033 (0.035)	0.346
Gender (29)	24.6 (21.2 – 28.4)[93]	-0.016 (0.011)	0.132
BMI (26)	23.8 (20.4 – 27.6)[92]	-0.066 (0.040)	0.096
Neck circumference (23)	25.5 (21.8 – 29.6)[92]	-0.289 (0.101)	0.004
Sample size			
> 200 (20)<br>< 200 (9)	23.5 (19.7 – 27.7)[94]<br>28.3 (18.7 – 40.4)[86]	-0.076 (0.325)	0.815
Study type			
Prospective (15)<br>Retrospective (8)<br>Cross sectional (6)	23.2 (17.7 – 29.8)[89]<br>26.0 (19.0 – 34.4)[95]<br>25.7 (18.0 – 35.1)[94]	0.247 (0.363)<br>-0.108 (0.345)	0.496<br>0.755
Validation tool			
Lab PSG (26)<br>HSAT (3)	25.2 (21.6 – 29.2)[93]<br>19.2 (7.0 – 42.9)[89]	-0.007 (0.556)	0.990
OSA criteria			
AHI ≥ 5 (28)<br>RDI ≥ 5 (1)	25.3 (21.8 – 29.1)[93]<br>10.5 (6.0 – 16.1)[-]	-1.719 (0.845)	0.042
Prevalence (29)	24.6 (21.2 – 28.4)[93]	0.014 (0.017)	0.428
North America (8)<br>South America (3)<br>Europe (6)<br>Middle East (6)<br>East Asia (3)<br>South/Southeast Asia (3)	20.2 (15.3 – 26.2)[88]<br>23.3 (15.8 – 32.8)[92]<br>22.2 (13.0 -35.3)[95]<br>32.0 (17.4 – 51.3)[94]<br>26.5 (18.1 – 37.0)[76]<br>32.4 (27.2 – 38.2)[25]	-1.185 (0.475)<br>-0.239 (0.555)<br>-0.659 (0.473)<br>-0.376 (0.488)<br>-0.123 (0.473)	0.013<br>0.667<br>0.164<br>0.441<br>0.796

AHI: Apnea-hypopnea index; BMI: Body mass index; CI: Confidence interval; HSAT: home sleep apnea testing; OSA: Obstructive sleep apnea; PSG: Polysomnogram; RDI: Respiratory disturbance index; SE: Standard error.

© 2021 Pivetta B et al. *JAMA Network Open.*

eTable 8. Test Characteristics of the Various STOP-Bang Score Cutoffs for the Different Severity of Apnea-Hypopnea Index in the Sleep Clinic Setting

STOP-Bang Score threshold	Number of studies	N	Sensitivity (%)	Specificity (%)	PPV (%)	NPV (%)
	**All OSA (AHI ≥ 5)**					
≥ 3	44	26540	89.2 (88.7-89.6)	40.6 (39.3-42.0)	85.6 (85.1-86.0)	48.1 (46.6-49.6)
≥ 4	12	5953	83.2 (82.1-84.2)	48.5 (45.7-51.4)	86.2 (85.2-87.2)	42.7 (40.0-45.3)
≥ 5	13	5523	64.1 (62.7-65.5)	65.8 (62.9-68.5)	87.8 (86.6-88.9)	32.3 (30.4-34.2)
≥ 6	9	4089	45.5 (43.7-47.2)	84.2 (81.6-86.5)	91.1 (89.6-92.4)	70.1 (69.0-71.3)
≥ 7	9	4089	17.5 (16.2-18.8)	95.2 (93.6-96.5)	92.8 (90.4-94.7)	24.5 (23.1-26.0)
≥ 8	8	3893	3.6 (3.0-4.3)	99.7 (99.0-99.9)	97.3 (91.7-99.3)	23.2 (21.9-24.6)
	**Moderate-to-severe OSA (AHI ≥ 15)**					
≥ 3	35	27629	93.0 (92.6-93.4)	32.1 (31.3-33.0)	64.7 (64.0-65.3)	77.5 (76.3-78.7)
≥ 4	10	5674	88.1 (86.9-89.2)	35.0 (33.1-36.9)	62.1 (60.7-63.6)	70.8 (68.2-73.2)
≥ 5	12	5360	67.0 (65.2-68.7)	59.5 (57.6-61.4)	65.9 (64.2-67.6)	61.8 (59.8-63.7)
≥ 6	9	3976	45.2 (43.0-47.3)	84.0 (82.3-85.6)	74.9 (72.4-77.3)	59.1 (57.2-60.9)
≥ 7	9	3977	20.3 (18.5-22.1)	95.0 (93.9-96.0)	81.2 (77.5-84.5)	52.8 (51.2-54.5)
≥ 8	8	4044	3.5 (2.7-4.4)	99.6 (99.2-99.9)	91.5 (81.9-96.5)	51.1 (49.5-52.6)
	**Severe OSA (AHI ≥ 30)**					
≥ 3	28	19972	95.9 (95.4-96.3)	26.9 (26.1-27.7)	45.0 (44.2-45.8)	91.4 (90.4-92.3)
≥ 4	6	2275	92.4 (90.2-94.2)	37.7 (35.3-40.2)	41.4 (39.0-43.8)	91.2 (88.7-93.2)
≥ 5	7	2486	77.2 (74.1-80.2)	54.5 (52.2-56.8)	41.4 (38.8-44.0)	84.0 (81.6-86.1)
≥ 6	5	2164	54.3 (50.5-58.0)	80.5 (78.4-82.5)	57.3 (53.5-61.1)	80.0 (77.8-82.0)
≥ 7	5	2192	25.4 (22.2-28.7)	93.8 (92.4-95.0)	66.3 (60.3-71.8)	72.3 (70.3-74.3)
≥ 8	5	2063	6.3 (4.5-8.5)	98.7 (97.8-99.3)	75.5 (61.4-85.8)	66.5 (64.4-68.6)

AHI: Apnea-hypopnea index; NPV: Negative predictive value; OSA: Obstructive sleep apnea; PPV: Positive predictive value.

© 2021 Pivetta B et al. *JAMA Network Open.*
